# Basic Limonoid modulates Chaperone-mediated Proteostasis and dissolve Tau fibrils

**DOI:** 10.1038/s41598-020-60773-1

**Published:** 2020-03-04

**Authors:** Nalini Vijay Gorantla, Rashmi Das, Hariharakrishnan Chidambaram, Tushar Dubey, Fayaj A. Mulani, Hirekodathakallu V. Thulasiram, Subashchandrabose Chinnathambi

**Affiliations:** 1 Neurobiology Group, Division of Biochemical Sciences, CSIR-National Chemical Laboratory, Dr. Homi Bhabha Road, 411008 Pune, India; 2 Division of Organic Chemistry, CSIR-National Chemical Laboratory, Dr. Homi Bhabha Road, 411008 Pune, India; 3 Academy of Scientific and Innovative Research (AcSIR), 411008 Pune, India

**Keywords:** Biochemistry, Biological techniques, Biophysics, Cell biology, Drug discovery, Neuroscience, Structural biology

## Abstract

The Alzheimer’s disease pathology is associated with accumulation of intracellular neurofibrillary tangles and extracellular senile plaques. The formation of initial nucleus triggers conformational changes in Tau and leads to its deposition. Hence, there is a need to eliminate these toxic proteins for proper functioning of neuronal cells. In this aspect, we screened the effect of basic limonoids such as gedunin, epoxyazadiradione, azadirone and azadiradione on inhibiting Tau aggregation as well as disintegration of induced Tau aggregates. It was observed that these basic limonoids effectively prevented aggregates formation by Tau and also exhibited the property of destabilizing matured Tau aggregates. The molecular docking analysis suggests that the basic limonoids interact with hexapeptide regions of aggregated Tau. Although these limonoids caused the conformational changes in Tau to β-sheet structure, the cytological studies indicate that basic limonoids rescued cell death. The dual role of limonoids in Tau aggregation inhibition and disintegration of matured aggregates suggests them to be potent molecules in overcoming Tau pathology. Further, their origin from a medicinally important plant neem, which known to possess remarkable biological activities was also found to play protective role in HEK293T cells. Basic limonoids were non-toxic to HEK293T cells and also aided in activation of HSF1 by inducing its accumulation in nucleus. Western blotting and immunofluorescence studies showed that HSF1 in downstream increased the transcription of Hsp70 thus, aggravating cytosolic Hsp70 levels that can channel clearance of aberrant Tau. All these results mark basic limonoids as potential therapeutic natural products.

## Introduction

Alzheimer’s disease (AD) is a progressive neurodegenerative disorder that affects the brain region associated with memory and cognitive functions. AD is caused due to deterioration of two key proteins essential for neuronal function, trans-membrane Amyloid-β and cytosolic Tau protein. Amyloid-β generated from Amyloid precursor protein is known to involve in cell adhesion, synaptogenesis, neuronal growth and development *etc*.^1–3^. The major function of Tau protein is to stabilize microtubule and is distributed predominantly in axons^4^. The pathological state is consequence of cellular insults such as abnormal activity of kinases and proteases, other post-translational modifications (PTMs) such as phosphorylation, acetylation, glycation, methylation *etc*. and oxidative stress^5^. In pathological conditions, Tau loses its affinity for microtubules and self-assembles into neurofibrillary tangles (NFTs). This leads to microtubule disassembly and loss of axonal integrity that ultimately cause neuronal death. To overcome the multifaceted triggers of AD pathology, molecules were screened from various origin including secondary plant metabolites^6,7^, synthetic compounds such as, peptides, methylene blue^8^, erythrosine B^9^, cyanine dye^10^ thiohydantoin and rhodanine derivatives^11–13^. Many of these molecules were found to have potent effect in overcoming Tau Pathology. Previously, several small molecules were screened for their contributions in preventing neurodegenerative disorders. In this concern, the structure-based docking and virtual screening has a significant role in the screening of small molecules^14^. But, since Tau lack rigid structure, the docking studies were carried out with Tau models for repeat regions^15^. The hexapeptides regions of Tau (^275^VQIINK^280^ and ^306^VQIVYK^311^), which forms β-sheets and promote self-aggregation was used to screen the small molecules for aggregation inhibition^16^. Resveratrol, a polyphenolic compound found abundantly in grape skin, led to the formation of Amyloid-β aggregates that are not toxic and lack definite structure^17^. Resveratrol was also found to modulate AD pathology by protecting against neuronal damage, excitotoxicity, rescuing the cells from oxidative damage and reducing neuro-inflammation^18,19^. EGCG, an active ingredient of green tea, is known to have anti-oxidant property. Studies in transgenic mice showed that administration of EGCG increased the activity of α-secretase, thus producing soluble Amyloid-β and decreasing the load of Amyloid-β in cortex and hippocampus^20,21^. Olive oil is also enriched with several polyphenolic compounds such as oleocanthal, oleuropein and its derivatives. These olive oil extracts effectively inhibited Tau aggregation when compared to methylene blue under *in vitro* conditions^6,22,23^. The aglycone form of oleuropein prevented Amyloid-β aggregation into soluble oligomers and thereby inhibiting its fibrillization^24^. Oleocanthal also exhibited similar properties and increased the clearance of Amyloid-β form brain^25^. Similarly, other phenolic compounds such as quercetin, apocynin and ellagic acid are reported to have a protective role in preventing AD^22,26–28^. As mentioned above, the clinical importance of extracts and purified metabolites from *Azadirachta indica* were well studied in various ailments, but their beneficial role in AD is not explored yet. Basic limonoids inhibit Tau aggregation and were non-toxic in HEK293T cells. The toxicity of resultant species obtained after the assay suggest that epoxyazadiradione-derived Tau species were non-toxic whereas the gedunin, azadirone and azadiradione-derived Tau species elicited moderate toxicity. AD is a neurodegenerative disorders depicted by proteotoxicity resulted due to imbalance in proteostasis network^29^. Such proteotoxic stress leads to the activation of heat shock transcription factor 1 (HSF1)^30,31^. However, levels of HSF1 are compromised during neurodegenerative diseases thus, alleviating Hsp70 levels and preventing the clearance of aberrant protein^32^. The basic limonoids, epoxyazadiradione induced the activation of HSF1 and increased the Hsp70 levels. Epoxyazadiradione, in contrast to Tau stress, effectively activated HSF1 as observed by accumulation of HSF1 in nucleus where it acts as transcription factor for Hsp70 expression. In our present study, we screened basic limonoids isolated from *Azadirachta indica* and analysed their effect in preventing Tau aggregation *in vitro*. Apart from their potent anti-feedant activities, limonoids are known to possess other biological activities such as anti-tumour, anti-inflammatory, anti-microbial, anti-diabetic, anti-oxidant and bone regeneration *etc*.^33,34^. Here, we analysed their mode of action in Tau aggregation inhibition and disaggregation of matured Tau aggregates; and studied the cytotoxicity effect of basic limonoids.

## Methods

### Chemical and reagents

MES, heparin, BES, BSA, BCA, CuSO_4_, ThS and ANS were purchased from Sigma. IPTG and DTT were purchased from Calbiochem. Other chemicals such as ampicillin, NaCl, KCl, Na_2_HPO_4_, KH_2_PO_4_, EGTA, MgCl_2_, PMSF, ammonium acetate were from MP and protease inhibitor cocktail was from Rosche. Dulbecco modified Eagle’s media (DMEM), Fetal Bovine Serum (FBS), Penicillin-Streptomycin were purchased from Gibco (Thermo fisher Scientific), and MTT (Thiazolyl blue tetrazolium bromide) was purchased from Sigma. The basic limonoids used here were isolated from fruit of *A. indica*. These compounds were characterized and well matched with that of reported data^35^.

### Expression and purification of Tau

The full-length and four repeat Tau was expressed in *E. coli* and the purification steps were carried out as described previously^36^. Tau expression was induced by 0.5 mM IPTG after the OD at A_600_ reached 0.5 to 0.6 and the cells were further grown for four hours before harvesting. The cells were harvested by pelleting at 4,000 rpm, for 10 minutes at 4 °C. The cell pellet was then resuspended in cell lysis buffer composed of 50 mM MES at pH 6.8, 1 mM EGTA, 2 mM MgCl_2_, 5 mM DTT, 1 mM PMSF and protease inhibitor cocktail. The cells were lysed at 15,000 psi by using constant cell disruption system. The lysate was then centrifuged at 40,000 rpm for 45 minutes at 4 °C. Thus, obtained supernatant was subjected to dialysis in Buffer A, containing 50 mM NaCl. The protein was the purified by increasing the NaCl gradient to 1000 mM. The obtained fractions were then analysed by SDS-PAGE, pooled and passed through size-exclusion chromatography. The protein concentration was estimated by bichinchoninic acid (BCA) method.

### Tau aggregation inhibition assay

The analysis of limonoids was carried out on Tau aggregates prepared *in vitro*. The soluble full-length Tau was incubated with heparin (17,500 Da) in the ratio of 4:1, in assembly buffer composed of 20 mM BES at pH 7.4, 25 mM NaCl, 1 mM DTT, 0.01% NaN_3_ and protease inhibitor cocktail. This mixture was incubated at 37 °C for the formation of matured aggregates. Further, the inhibition of Tau aggregation in presence of limonoids was studied by incubating 20 µM Tau with 400 µM of limonoids.

### Disaggregation assay

The ability of limonoids to disintegrate the mature Tau aggregates were analysed by incubating 20 µM of preformed Tau aggregates with 400 µM of limonoids. The extent of disaggregation was analysed by ThS and ANS fluorescence assay and the change in aggregates morphology was observed by TEM.

### Molecular docking studies

The following four limonoids, azadirone, azadiradione, epoxyazadiradione and gedunin were used as ligands. Ligand structures were accessed from the ChemSpider database, and the 3D coordinates of ligands were generated using OpenBabel^37,38^. DFT calculations were carried out to optimize the ligands prior to their use in docking, using Gaussian 09 program^39^ by employing B3LYP/3–21G^40^ level of theory. The cryo-electron microscopy structure of aggregated Tau filaments with a resolution of 3.3 Å was accessed from the RCSB-PDB repository^41,42^. (PDB ID. 6QJH) The initial protein preparation for docking analysis was performed on PyMol^43^, and the Tau fragment (Gly272 to His330) was structure refined by the GalaxyWEB server^44,45^. The structure refined model of Tau fragment was used as the receptor. The ligands and the aggregated Tau model were prepared for docking using AutoDock Tools 1.5.6^46^. The hydrogen atoms were added to the protein, and then the grid parameter file (gpf) was generated. The ligand molecules were added with gasteiger charges, the non-polar hydrogen atoms were merged, and all active bonds were made non-rotatable. The Lamarckian Genetic Algorithm was used to generate the docking parameter file. (dpf) Docking of basic limonoids with the Tau protein was performed using AutoGrid4.2 and Autodock4.2^47^. The docked protein-ligand complex were visualized using PyMol^43^, and interaction studies are performed using LigPlot+ 2.1^48,49^. The corresponding 3D representation of the interactions were visualized using PyMol^43^.

### Fluorescence assay

The native Tau during aggregation attains β-sheet structure and this was probed by Thioflavin S (ThS) binding. The increase in ThS fluorescence is the characteristics of Tau assembly into PHFs^50^. 5 µL of Tau from reaction mixture was diluted to 50 µL with 8 µM ThS prepared in 50 mM ammonium acetate, pH 7.0. The assay was carried out in triplicates in 384 black well plates. The plate was incubated in dark for 10 minutes. The fluorescence spectrum was recorded at 521 nm by exciting ThS at 440 nm in Tecan Infinite 200 PRO multimode micro plate reader.

### ANS fluorescence assay

Similarly, the hydrophobicity also gradually increased during Tau aggregation. The change in hydrophobicity was analysed by ANS (8-Anilinonaphthalene-1-sulfonic acid). The intensity of this fluorescent probe increased upon interacting with hydrophobic regions in Tau aggregates. The ANS fluorescence was measured by incubating 5 µL of Tau from reaction mixture with 40 µM ANS prepared in 50 mM ammonium acetate, pH 7.0. The ANS fluorescence assay was carried out similarly as ThS fluorescence assay by exciting ANS at 390 nm and recording the emission at 475 nm in Tecan Infinite 200 PRO multimode micro plate reader.

### Sedimentation assay and SDS-PAGE analysis

The formation of higher order aggregates by Tau in presence and absence of limonoids were analysed by SDS-PAGE. In sedimentation assay Tau was subjected to centrifugation at 60,000 rpm for 1 hour. After centrifugation, the supernatant was collected and labelled as supernatant (S). The pellet formed was resuspended in equal volumes of buffer and labelled as pellet (P). The reaction mixture that was not centrifuged was labelled as total (T). Thus, obtained supernatant, pellet and total fractions were analysed by SDS-PAGE at time points of 0, 24 and 120 hours. The full-length Tau was resolved by using miniVE Vertical Electrophoresis System purchased from GE Healthcare Life Sciences and the repeat Tau was resolved by Mini-PROTEAN Tetra Vertical Electrophoresis purchased from Bio-Rad.

### CD spectroscopy

The conformational changes in Tau was analysed by CD spectroscopy in the far-UV region. It details the change in native conformation of Tau *ie*., random coil to β-sheet structure, which signifies Tau aggregation. The spectra were recorded by using Jasco J-815 spectroscopy as described previously^51^. The sample was diluted to 3 µM in phosphate buffer at pH 6.8 and the measurements were carried out in 1 mm path length cuvette by adjusting the wavelength to a range from 250 nm to 190 nm. The spectra were measured at 25 °C, with data pitch of 1.0 nm, scanning speed of 100 nm/min, data integration time of 1 second and 6 accumulations were recorded for each sample. The spectrum was plotted using SigmaPlot 10 software.

### Transmission electron microscopy (TEM)

The tendency of Tau to assemble and form aggregates led us to analyse their morphology by TEM. Tau was diluted to a final concentration of 1 µM and spotted on copper coated carbon grids purchased from Ted Pella, Inc. An electron dense layer was provided to Tau aggregates by staining with 2% uranyl acetate. The grid was dried and analysed by Tecnai T20 at 200 kV at magnification of 3500×, 2250×, 8700× and 6200× that corresponds to the scale bar of 0.2 μm, 0.5 μm, 100 nm and 200 nm respectively.

### Filter trap assay

Tau protein incubated along with the limonoids at the concentration of 400 µM was filtered across nitrocellulose (NC) membrane by applying vacuum. The blot was then incubated for 1 hour with the blocking buffer, containing 5% skimmed milk in PBST. Further, the blot was treated with K9JA antibody that was diluted in the ratio of 1:8000. Primary antibody was diluted in blocking buffer and the blot was probed for 1 hour at room temperature. The blot was washed with PBST thrice for 10 minutes each. The secondary antibody *i.e*., goat anti-rabbit HRP conjugated IgG against K9JA was added in 1:10000 dilution and probed for 1 hour. Again the blot was washed with PBST thrice, 10 minutes each and transferred in PBS. The blot was developed by ECL (Enhanced Chemiluminisence) reagent and chemiluminisence was recorder by Amersham Imager 600.

### Preparation of tau aggregates for cytotoxic assay

The full-length Tau was expressed in *E. coli* strain BL21* and the purification was done according to the laboratory protocol. 100 μM of Tau in 20 mM BES pH 7.4, 25 mM NaCl was started with 25 μM of heparin (17.5 kDa) and was incubated at 37 °C for 7 days. The formation of aggregates were analysed by ThS fluorescence, SDS-PAGE and TEM.

### Cytotoxicity assay

HEK293T cells (ATCC CRL-11268) were cultured in DMEM (Dulbecco modified eagle’s media) with 10% FBS and 100 μg/mL of penicillin and streptomycin. 10^4^ cells/well were seeded in 96-well plates and incubated at 37 °C for 12 hours at 5% CO_2_. Basic limonoids (gedunin, azadirone, azadiradione and epoxyazadiradione) were added at a range from nano molar to micro molar concentrations, with 5% DMSO as a control at final volume of 100 μL and incubated for 24 hours at 37 °C, 5% CO_2_. After 24 hours, MTT (Thiazolyl blue tetrazolium bromide) reagent was added at a concentration of 0.5 mg/mL in each well and incubated for 3 hours at 37 °C, 5% CO_2_. Adding DMSO solubilized the formazan end product and the absorption was measured at 570 nm in spectrophotometer (Tecan Infinite 200 PRO multimode micro plate reader).

### Cytotoxicity inhibition assay by basic limonoids

HEK293T cells at a density of 10^4^ cells/well were seeded in 96-well plates and incubated at 37 °C for 12 hours, 5% CO_2_. The preformed human Tau aggregates at a concentration of 10 μM were added in each well and the basic limonoid compounds (gedunin, azadirone, azadiradione and epoxyazadiradione) at 1, 2, 5, 10, 20, 50 μM concentrations were added with only human Tau aggregates as a negative control at final volume of 100 μL and incubated for 24 hours at 37 °C, 5% CO_2_. After 24 hours, MTT reagent was added at a concentration of 0.5 mg/mL per well and incubated for 3 hours at 37 °C, 5% CO_2._ Adding DMSO solubilized the formazan and the absorption at 570 nm measured in spectrophotometer (Tecan Infinite 200 PRO multimode micro plate reader). Limonoids effectively prevent Tau aggregation and thus led to the formation of distinct Tau species as observed by TEM. The toxicity of the resultant Tau species were studied by MTT assay, where 10,000 cells were seeded per well. After 24 hours, cells were treated with the reaction mixture at 5 μM Tau concentration. MTT was added at a concentration of 500 μg/mL post-incubation for 24 hours. After addition of MTT the cells were further incubated for 3 hours. The resultant formazan is dissolved by adding 100 μL DMSO. The obtained purple colour complex is read at 570 nm in spectrophotometer (Tecan Infinite 200 PRO multimode micro plate reader).

### Western blotting

The effect of epoxyazadiradione on levels of HSF1 and Hsp70, with actin as control was studied using western blotting. HEK293T cells were plated in 6 well culture plates as 3 × 10^6^ cells/well. The cells were grouped into 4 experimental groups *viz*. cell control, cells stressed with 1 μM Tau aggregates, 10 μM epoxyazadiradione and cells stressed with Tau aggregates in presence of epoxyazadiradione. After 24 hours of incubation cells were lysed in RIPA buffer (10 mM Tris-Cl pH 8.0, 0.5 mM EGTA, 1% Triton X 100, 0.1% sodium deoxycholate, 0.1% SDS, 140 mM NaCl and protease inhibitor cocktail) followed by SDS-PAGE and transferring the protein onto PVDF membrane. The membrane was subjected to blocking using 5% milk for 1 hour at room temperature. The blots were incubated separately with rabbit polyclonal anti-HSF1 (Thermo Fisher; PA3-017) and mouse monoclonal anti-Hsp70 (Thermo Fisher; MA3-006) at a dilution of 1:2000 and 1:1000 respectively, overnight. The blots were then washed thrice with 1X PBST and incubated with HRP conjugated goat anti-rabbit and goat anti-mouse secondary antibodies at a respective dilution of 1:10000 and 1:1500. Blots were subsequently washed with 1X PBST thrice before transferring into 1X PBS. The blot was stripped and incubated with rabbit polyclonal anti-α-actin (Sigma; A2066) at dilution of 1:1500 overnight. The blot was then washed with 1X PBST, followed by incubation with goat anti-rabbit at dilution of 1:10000, subsequently the blot was washed and developed. The blot was developed by using Clarity western ECL blotting substrates from Bio-Rad. The blots were imaged in Amersham imager 600.

### Immunofluorescence

HEK293T cells were passaged in advanced DMEM, supplemented with 10% FBS and 100 μg/mL penicillin-streptomycin. In order to check the functionality of HSF1 in epoxyazadiradione treated Tau-stressed cells, HEK293T cells (25,000 cells/well) were seeded onto the coverslip and treated with Tau (1 μM) and epoxyazadiradione (50 μM) separately and simultaneously for 24 hours. After incubation, the cells were washed twice with PBS and fixed with chilled methanol for 10 minutes. The cells were permeabilized and blocked with 0.2% Triton-X 100 and 5% horse serum respectively. Then, HEK293T cells were immunostained with HSF1 (1:50) and Tau T46 (Thermo; 13–6400, 1:250) antibody along with counterstain DAPI. To understand the potential of extracellular Tau aggregates as a triggering factor for cellular proteostasis pathway, HEK293T cells were treated with Tau and epoxyazadiradione; immunostained with HSF1 (1:50) and Hsp70 (1:100) antibody in four treatment groups (cell control, Tau, epoxyazadiradione, Tau+epoxyazadiradione) as described previously. The subcellular localization of HSF1 were analysed by Zeiss Axio observer microscope 7 with apotome 2.0 and the imaging was done using 63X oil immersion objective lens. The intensity of HSF1 and Tau levels from each cells were quantified by Zen 2.3 software (https://www.zeiss.com/microscopy/int/products/light-microscopes/axio-observer-for-biology.html) in multiple fields (n = 10) and mean intensity per area were calculated for various treatment groups. The nuclear *vs*. cytosolic concentration of HSF1 were quantified by Zen 2.3 software in multiple fields (n = 10) and mean intensity per area were plotted for four treatment groups.

### Statistical analysis

All experiments were carried for two biological replicates. Statistical analyses were performed for microscopic quantification in each treatment groups (n = 7) by using two-tailed Student’s *t*-test using SigmaPlot 10.0, from Systat Software, Inc., San Jose California USA, www.systatsoftware.com. Values were represented as Mean + SD. *Corresponds to test groups compared with cell control (*p < 0.05; **p < 0.01, ***p < 0.001, ns: non-significant) and ^#^corresponds to test groups compared with Tau treated group (^#^p < 0.05; ^##^p < 0.01, ^###^p < 0.001, ns: non-significant).

## Results

### Basic limonoids inhibits Tau aggregation *in vitro*

**Figure 1:**
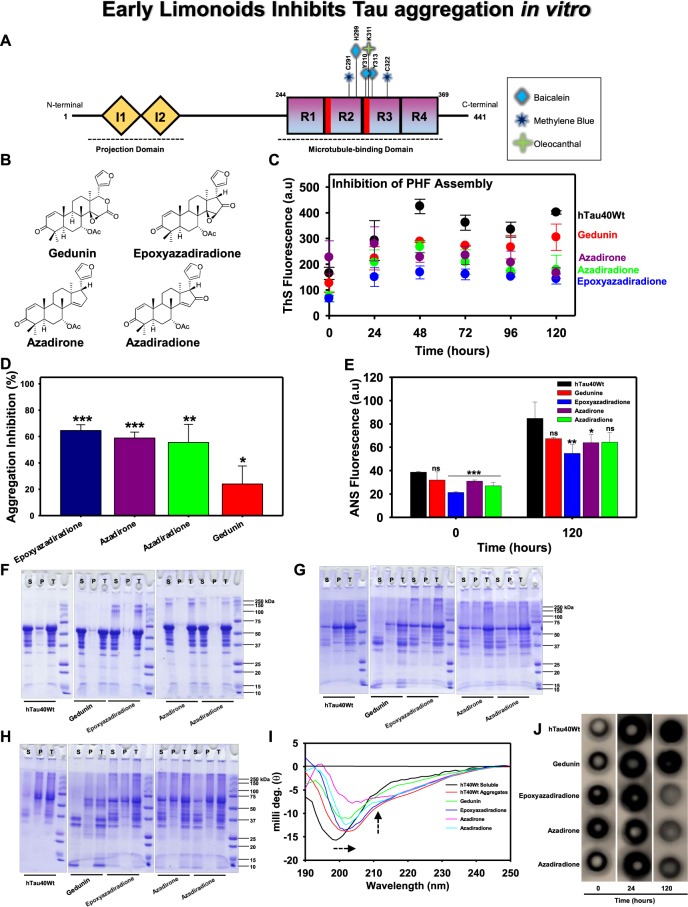
The aggregation inhibition of Tau by basic limonoids. (**A**) Diagrammatic representation of full-length Tau comprising 441 amino acids with two inserts towards N-terminal and four repeats towards C-terminal. The inserts functions as projection domain, which serves to separate microtubule bundles. The four repeats act as microtubule-binding domain and helps in tubulin polymerization into microtubules. AD triggers pathological modifications in repeat region, leading to the loss of Tau affinity for microtubule. This causes destabilization of microtubule and aggregation of Tau. The bar diagram depicts the binding sites for various small molecules such as Baicalein, Methylene blue and Oleocanthal interacting with Tau. (**B)** Structure of the basic limonoids isolated from the fruit coat of *Azadirachta indica*. (**C,D)** Inhibition of full-length Tau aggregation by basic limonoids was analysed by ThS fluorescence assay which shows the potency of epoxyazadiradione over other limonoids, which was also represented as rate of inhibition in terms of percentage. (**E**) ANS fluorescence revealed the change of hydrophobicity content in Tau at time intervals of 0 and 120 hours, according to which epoxyazadiradione have persistently low hydrophobicity when compared to control and other limonoids. (**F)** The SDS-PAGE analysis showed the formation of higher order aggregates at 0 hour by epoxyazadiradione, azadirone and azadiradione, whereas full-length Tau, the control shows no signature of aggregation. The sedimentation assay revealed the presence of these aggregates in supernatant (S) and not in the pellet (P) which indicated that these were soluble aggregates. (**G)** After 24 hours of incubation the full-length Tau aggregated and this phenomenon was observed in control and test. This was witnessed by Tau present in the pellet (P) fraction obtained after sedimentation. Tau exhibited extensive degradation in presence of gedunin, whereas other limonoids showed Tau aggregation. (**H)** At 120 hours the Tau was completely aggregated and separated in the pellet (P). Gedunin exhibited mostly the degradation of soluble Tau although aggregation was also evidenced. Epoxyazadiradione, azadirone and azadiradione also exhibited Tau aggregation at varied extent. (**I)** The CD spectra of native Tau showed random coil conformation, whereas on aggregation the spectra signified β-sheet conformation. Similarly, in presence of limonoids the change in Tau conformation to β-sheet was observed. The basic limonoids exhibited difference in the spectrum intensity and shift. This could be due to the variation in the structure of limonoids which led Tau to attain varied conformations. (**J)** The aggregation of Tau was probed by K9JA reveals the initial aggregation by limonoids at 0 and 24 hours. Later at 120 hours of incubation shows the fully aggregated Tau in absence of limonoids. Epoxyazadiradione showed more potential in inhibiting aggregation, followed by Azadirone and Azadiradione. Gedunin had poor effects in preventing aggregates formation. The graph was plotted using SigmaPlot 10.0, from Systat Software, Inc., San Jose California USA, www.systatsoftware.com.

The characteristics of pathologically modified Tau to assemble and aggregate leads to AD, a neurological disease that is associated with loss of memory and cognitive functions. The full-length isoform of Tau is composed of 441 amino acids with two inserts and four repeats that play a key role in neuronal physiology and pathology of AD (Fig. 1A). In the present study, the role of four different basic limonoids isolated from the fruit of *Azadirachta indica* were analysed against Tau aggregation (Fig. 1B). *In vitro* conditions the aggregation of Tau was induced by heparin, resulting in two signature features, β-sheet structures and hydrophobic patches as analyzed by Thioflavin S (ThS) fluorescence and 8-Anilinonaphthalene-1-sulfonic acid (ANS) respectively. *In vitro* aggregation of full-length Tau was witnessed by gradual increase in ThS fluorescence (Fig. 1C). Decrease in fluorescence intensity in presence of limonoids attributing to inhibition of aggregation was seen (Fig. 1D). Amongst the limonoids screened, epoxyazadiradione exhibited the highest inhibition of 63.5% followed by, azadirone and azadiradione resulting in 57.6% and 54.2% respectively. The lowest inhibition of 21.7% was exhibited by gedunin on Tau aggregation. Further, the analysis of hydrophobicity in Tau aggregates by ANS manifested epoxyazadiradione to effectively reduce the hydrophobicity whereas, gedunin, azadirone and azadiradione, exhibited similar proportion of Tau hydrophobicity in solution (Fig. 1E). Further the extent of aggregation in presence and absence of limonoids were analysed by SDS-PAGE. The full-length Tau showed no signature of aggregation at 0 hour (Fig. 1F). However, upon sedimentation, it was evidenced that epoxyazadiradione, azadirone and azadiradione formed higher order aggregates. At the end of 24 hours, the protein was separated in pellet (P) and this suggested the aggregation of Tau (Fig. 1G). In presence of gedunin, the soluble Tau in supernatant (S) was degraded and some proportion of Tau was also known to aggregate. Hence, indicating that gedunin serves to prevent aggregation by enhancing degradation of Tau. Furthermore at 120 hours, Tau was completely aggregated, but epoxyazadiradione, azadirone and azadiradione showed different rates of aggregation, which was evidenced by the separation of Tau in supernatant and pellet after sedimentation (Fig. 1H). Gedunin at this time point completely degraded the soluble Tau and it barely showed Tau aggregation. This effect of gedunin on Tau aggregation was distinct when compared to other basic limonoids. The extent of aggregation by Tau was also analysed by filter trap assay (Fig. 1J). Tau was probed by K9JA antibody, which indicate that limonoids were forming aggregates at 0 and 24 hours but, it was prevented at 120 hours. Tau showed aggregation at 120 hours in absence of limonoids. However, gedunin had negligible effect in preventing Tau aggregation as evidenced by Thioflavin S fluorescence assay.

### Conformational changes of full-length Tau by basic limonoids

In native conditions, Tau adopts random coil conformation and is devoid of secondary structure. However, Tau attains β-sheet conformation after aggregation and this change in conformation was analysed by CD spectroscopy. The differential absorption spectra of protein in far-UV region were measured which revealed change in Tau conformation. Tau in its native conformation absorbs at 190 nm and the absorbance shifts to a higher wavelength with bathochromic effect upon aggregation. In presence of limonoids Tau showed β-sheet conformation, which was a significant pattern followed by all the basic limonoids (Fig. 1I). Epoxyazadiradione exhibited fewer shift in ellipticity, followed by azadiradione, gedunin and azadiradione. This indicates that when compared to other limonoids, epoxyazadiradione played effective role in preventing change in Tau conformation. Further, the peak intensity reflected the hydrophobicity in Tau and it revealed that in presence of epoxyazadiradione Tau exhibited less hydrophobicity. Further, the decrease in ellipticity by azadirone indicates the change in Tau structure when compared to other limonoids.

### Fragility of Tau aggregates induced by limonoids

**Figure 2:**
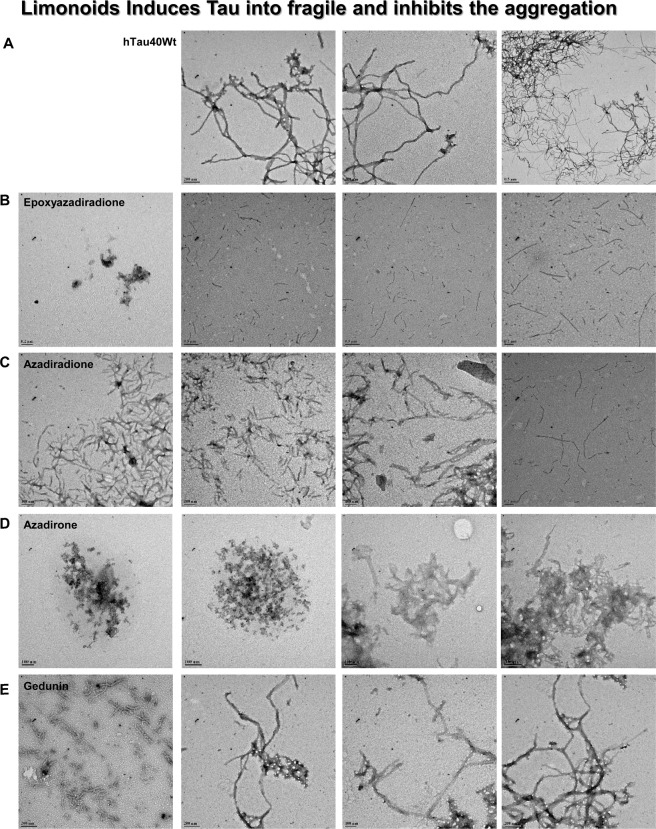
TEM analysis for Tau aggregation in presence of basic limonoids. (**A)** The negative staining of Tau aggregates are formed *in vitro* in presence of inducer exhibited polymorphic structure of Tau morphology. (**B,C)** Epoxyazadiradione and azadiradione inhibited Tau aggregation and limited the Tau aggregation to shorter length filaments. (**D)** Azadirone led to the formation of amorphous aggregates by Tau. (**E)** Tau aggregates exhibited change in morphology and twist in presence of gedunin. This result indicated that gedunin might have altered the mode of aggregation, but have no effect on rate of aggregation.

Tau aggregates exhibit polymorphic structure that forms long and straight filaments with twisted morphology. In absence of limonoids Tau form extensive aggregates that are observed as long filaments by TEM (Fig. 2A). All the limonoids showed inhibition of Tau aggregation, although with varied morphologies. Epoxyazadiradione and azadiradione limits Tau aggregation to shorter lengths (Fig. 2B,C). In comparison with azadiradione, epoxyazadiradione showed much smaller aggregates. Besides, azadirone led to the formation of amorphous aggregates, which suggested that it prevents Tau assembly into matured filaments (Fig. 2D). Further in presence of gedunin, Tau exhibits the morphology of both short fragments as well as the extensively formed aggregates (Fig. 2E). These observations revealed that limonoids have varied effects on Tau aggregation, which could be due to the difference in their interaction with Tau.

### The repeat Tau inhibition modulated by basic limonoids

The aggregation inhibition potency of limonoids against repeat Tau was analyzed by fluorescence assay. For the assay, *in vitro* aggregation of repeat Tau was induced by heparin. The mixture was incubated with 400 µM of basic limonoids. The ThS fluorescence assay indicated that limonoids have poor inhibitory effect. Epoxyazadiradione followed by azadiradione exhibits greater inhibition amongst all the basic limonoids (Fig. S4A). The SDS-PAGE analysis evidenced the absence of higher molecular weight Tau in presence of limonoids (Fig. S4B). This observation is in comparison with control *ie*., in absence of limonoids. Further the effect of limonoids was addressed by sedimentation assay. It revealed that the repeat Tau was devoid of protein in pellet (P), which signifies the absence of aggregates at 0 hour. In presence of gedunin, epoxyazadiradione and azadirone lower levels of Tau were observed in pellet (Fig. S4C). This indicates the presence of higher order species. These species were absent in presence of azadiradione. This indicate that at 0 hour gedunin, epoxyazadiradione and azadirone were initially leading to the formation of higher order species, which are later either solubilised or restricted to mature into aggregates as analysed by TEM.

### Basic limonoids disintegrates the pre-formed fibrils

**Figure 3:**
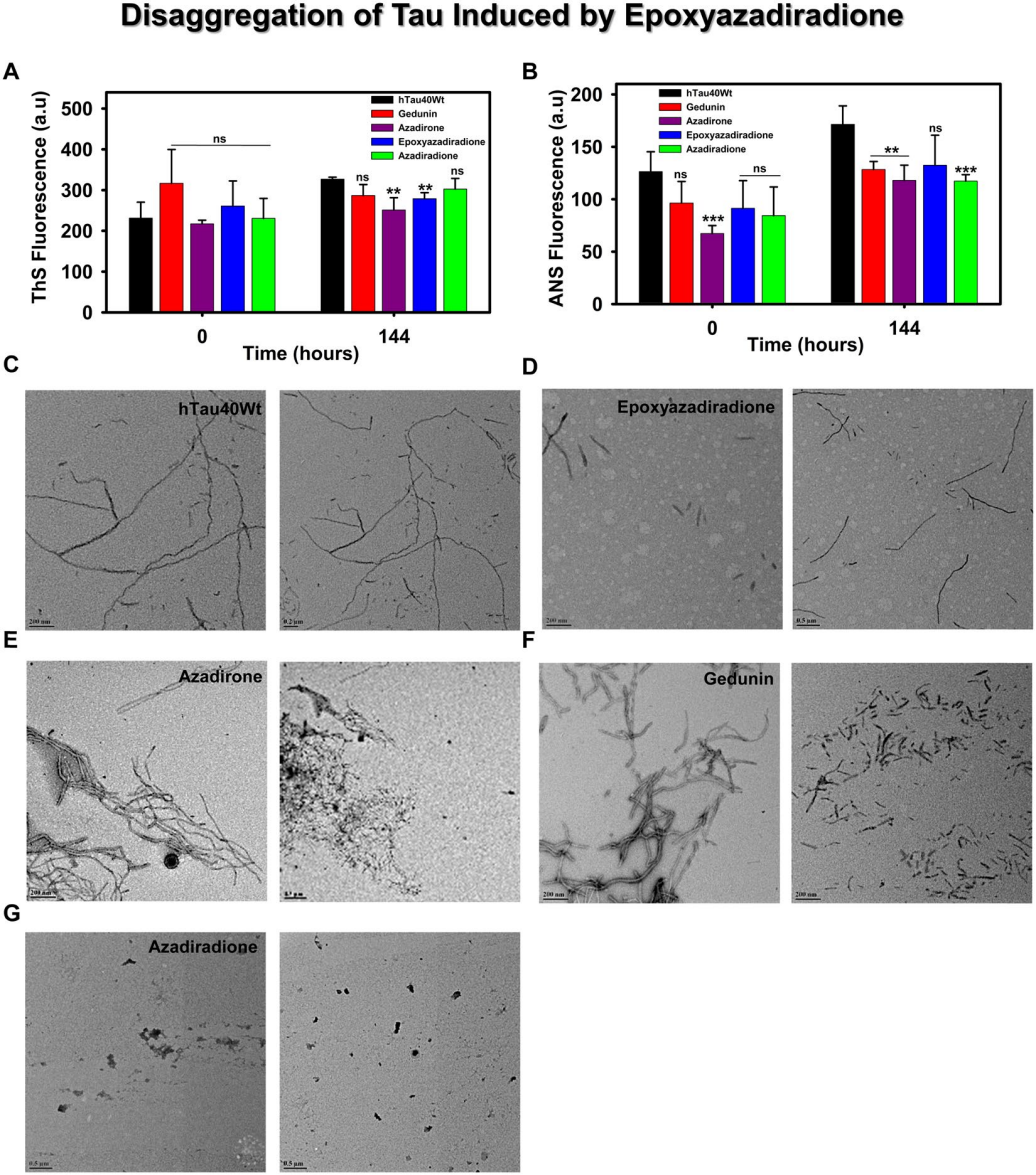
Disaggregation of pre-formed Tau aggregates by basic limonoids. (**A,B**) The role of basic limonoids in disaggregation of Tau was analysed by ThS and ANS fluorescence assay. The ThS and ANS fluorescence were evidenced to increase at 144 hours. This indicated that limonoids have no role in solubilizing the preformed Tau aggregates. (**C)** The aggregates of Tau exhibited the morphology of extended and thick filaments in the absence of limonoids. (**D)** Epoxyazadiradione destabilizes the pre-formed aggregates to shorter fragments. (**E,F)** Azadirone and gedunin altered the morphology of Tau aggregates and disintegrated them into shorter filaments. **(G)** Azadiradione completely destabilized Tau aggregates. The graph was plotted using SigmaPlot 10.0, from Systat Software, Inc., San Jose California USA, www.systatsoftware.com.

During AD, the affected region is populated by mature Tau aggregates, which are observed as plaque depositions^52^. It is also essential to clear such toxic accumulations for normal functioning of neuron. Hence, it was necessary to screen the compounds that cleared deposited aggregates. The varied role of limonoids in altering Tau aggregation providing a hint about its inhibitory property. Here, we studied their effect in destabilizing the preformed Tau aggregates. The disaggregation of Tau was probed by ThS and ANS fluorophores (Fig. 3A,B). It was observed that ThS showed no signs of disintegration but ANS showed increase in fluorescence, which represented increase in Tau hydrophobicity. The Tau aggregates analysed by TEM showed filamentous and thick aggregates in absence of limonoids (Fig. 3C). However, it suggested that each limonoids have different effects on Tau disintegration (Fig. 3D–G).

### Interaction study of basic limonoids with aggregated Tau by molecular docking

**Figure 4:**
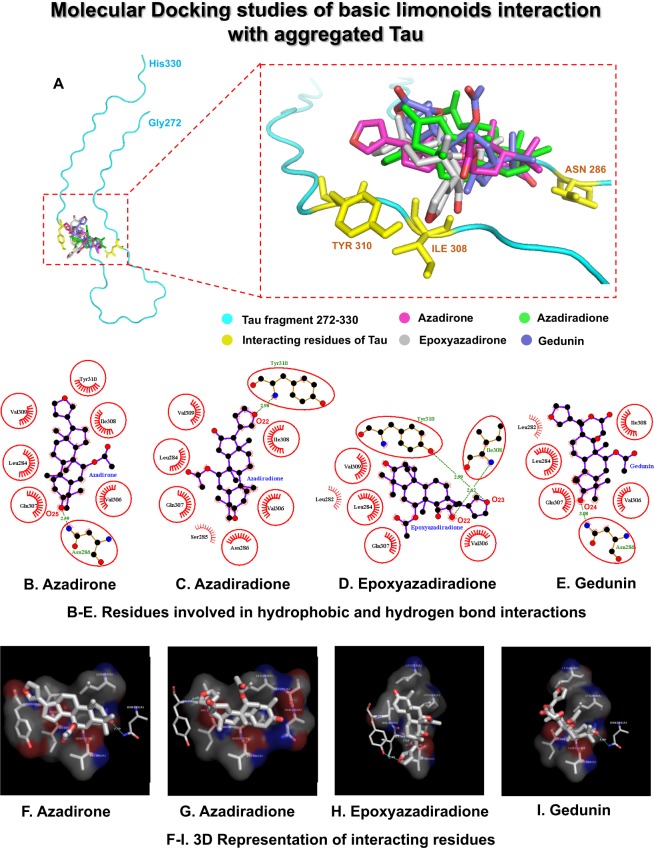
Molecular Docking studies of basic limonoids with aggregated Tau model (Gly272-His330). (**A)** shows the binding site for the ligands and the residues of Tau involved in hydrogen bond interactions. (**B–E**) represents the 2D-graph generated by LigPlot+ for hydrogen bond and hydrophobic interactions of the ligands with the Tau residues. (**B)** Azadirone, (**C)** Azadiradione, (**D)** Epoxyazadiradione, and (**E)** Gedunin. (**F–I)** represents the corresponding 3-dimensional view of the interaction visualized by PyMol. (**F**) Azadirone, (**G**) Azadiradione, (**H**) Epoxyazadiradione, and (**I**) Gedunin.

The molecular docking analysis was performed to study the role of limonoids in Tau aggregation inhibition and disintegration of matured aggregates. Zhang, *et al*.^42^ generated cryo-electron microscopy structures of heparin-induced recombinant full-length Tau (2N4R). Among them, the snake structure of Tau (Gly272 to His330) was reconstructed with an overall resolution of 3.3 Å and six β-sheets within the structure. This structure was refined and used as the model for our docking studies. The hexapeptide region of Tau (^306^VQIVYK^311^) plays a major role in nucleation dependent aggregation of monomeric Tau to filaments. Hence, in this study, the interaction of basic limonoids with the hexapeptide region was analysed. The structure refined Tau model (6QJH) had an RMSD value of 0.776 Å. The grid map generated for the hexapeptide region of aggregated Tau fragment had a grid point spacing of 0.375 Å with grid points of 88, 88, 68 and a grid center of approximately 130, 146, 200 on the X, Y, and Z-axis respectively. The ligands were structure optimized and the corresponding co-ordinate files are provided in the supplementary data (Fig. S10A–D). The molecular docking analysis revealed the best pose for each ligand on the grid generated for the hexapeptide region of Tau. The interacting structure for each ligand with the lowest binding energy was taken for analysis (Fig. 4A). The binding pocket was formed by Tau residues Leu282, Leu284, Ser285, Asn286, Val306, Gln307, Ile308, Val309 and Tyr310 (Fig. 4B–E). The corresponding 3-dimensional representation of Tau residues involved in hydrophobic and hydrogen bond interaction with the basic limonoids are shown in Fig. 4F–I. Epoxyazadiradione has the lowest binding energy value of −6.57 kcal/mol, followed by azadirone, azadiradione and gedunin with the binding energy values of −6.19, −5.89, and −5.21 respectively. In epoxyazadiradione, carbonyl oxygen atoms, O_22_ and O_23_ interact with the -NH group of Ile308 and the -OH group of Tyr310 with a hydrogen bond distance of 2.62 and 2.99 Å respectively (Fig. 4D and H). In azadirone, the O_25_ atom of carbonyl group interacts with the -NH_2_ group of Asn286 with a hydrogen bond distance of 2.59 Å (Fig. 4B and F). In azadiradione, the O_22_ atom of carbonyl group interact with the -NH group of Tyr310 with a hydrogen bond distance of 2.98 Å (Fig. 4C and G). In gedunin, the O_24_ atom of carbonyl group interacts with the -NH_2_ group of Asn286 (Fig. 4E and I) (Table 1). From this study, it can be observed that epoxyazadiradione has the lowest free energy of binding which indicates its higher affinity for Tau, followed by azadirone, azadiradione and gedunin in aggregation inhibition and disintegration of matured Tau aggregates. Hence, epoxyazadiradione could be a potent molecule in inhibiting Tau aggregation.

**Table 1:** Tau residues involved in hydrogen bond and hydrophobic interactions.

Interacting residues of Tau	Epoxy azadiradione −6.57 kcal/mol*	Azadirone −6.19 kcal/mol*	Azadiradione −5.89 kcal/mol*	Gedunin −5.21 kcal/mol*
Leu282		—	—	
Leu284				
Ser285	—	—		—
Asn286	—			
Val306				
Gin307				
lle308				
Val309				—
Tyr310				—

— Non-interacting residues  Tau residues with Hydrogen bond interactions  Tau residues with hydrophobic interactions *Mean binding energy of ligands.

### Basic limonoids inhibit the human Tau-mediated cytotoxicity

**Figure 5:**
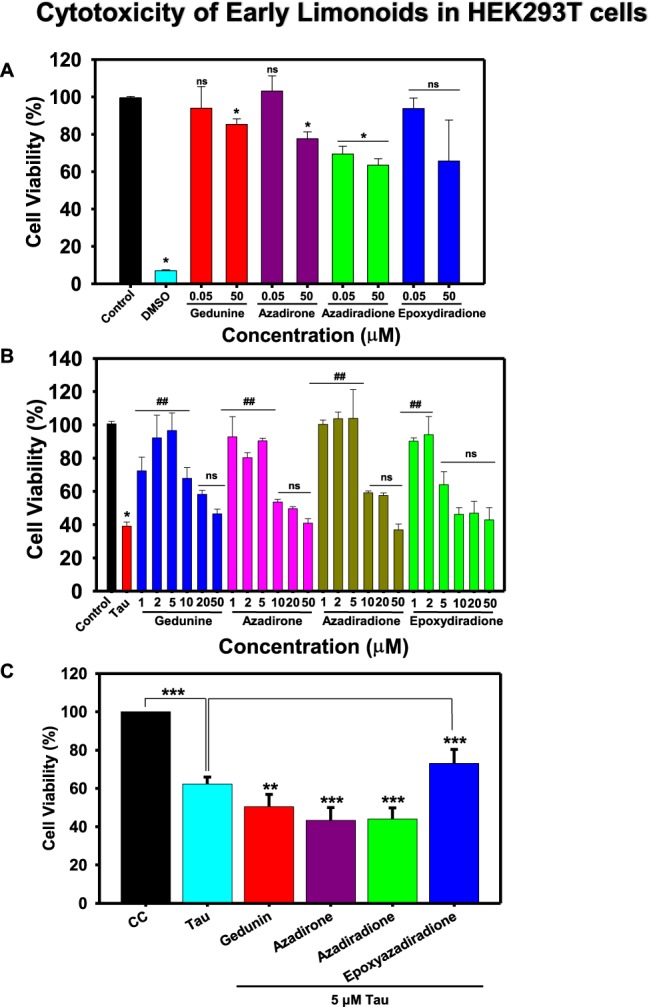
Cytotoxicity by basic limonoids on HEK293T cell line by MTT assay. (**A)** 10^4^ cells/well were seeded in 96 well plate and incubated with 0.05 and 50 μM concentrations of basic limonoids at 37 °C, 5% CO_2_ for 24 hours, followed by MTT assay and calculation of percentage cell viability. The toxicity profile for four basic limonoids were: azadiradione>epoxyazadiradione>gedunin>azadirone. (**B)** HEK293T cells were seeded in 96 well plate and co-treated with 10 μM of full-length Tau aggregates along with six different concentrations (1, 2, 5, 10, 20, and 50 μM) of the limonoids and incubated for 24 hours at 37 °C, 5% CO_2_. MTT Assay determined cell viability where azadiradione and epoxyazadiradione at 1 μM concentration were more effective to reduce human Tau aggregates mediated toxicity as compared to azadirone and gedunin in HEK293T cells. (**C)** The toxicity of resulted Tau species in presence and absence of basic limonoids were analysed by cell viability assay. The viability of Tau treated cells decreased to 62%, whereas in presence of epoxyazadiradione less toxic species were formed resulting in 73% viability. Gedunin, azadirone and azadiradione formed toxic Tau species due to which the cell viability was reduced to 50%, 43% and 44% respectively. The graph was plotted using SigmaPlot 10.0, from Systat Software, Inc., San Jose California USA, www.systatsoftware.com.

Neurodegenerative diseases were found to be associated with the cellular toxicity, ultimately leading to the neuronal death among, which Tau aggregates-mediated toxicity is one of the leading causes. Toxicity of limonoids and/or Tau were analysed on the basis of cell morphology (Figs. S1A–F, S2A–F). The preformed Tau aggregates when added extracellular to HEK293T cells, it showed 32% cellular viability at 10 μM Tau concentration. But, treating the cells with basic limonoids inhibited cytotoxicity. Azadiradione reduced the toxicity mediated by human Tau aggregates at a lower concentration of 1 μM. Further, azadirone at 5 μM concentration reduced 50% Tau toxicity whereas; gedunin and epoxyazadiradione showed 60% reduction in Tau toxicity at 5 and 2 μM concentration respectively. When the HEK293T cells were treated only with limonoids at 50 μM concentrations, gedunin, azadirone, epoxyazadiradione and azadiradione showed 84%, 78%, 64% and 61% cytotoxicity respectively. These results clearly suggest the potency of limonoids in Tau aggregation inhibition and dissolution of preformed fibrils in AD (Fig. 5A,B). The non-toxic nature of limonoids in HEK293T and their ability to reduce toxicity of Tau suggest them as potential molecules to prevent AD.

### Cytotoxicity of total Tau species

The cell viability assay showed the non-toxic nature of basic limonoids and can prevent Tau-mediated toxicity in HEK293T cells. We studied the effect of Tau species resulted upon incubation of Tau with basic limonoids. The toxicity of aggregated Tau in cells is a well-known phenomenon; in our present study we observed that Tau aggregates showed about 62% viability whereas, epoxyazadiradione treated Tau resulted in 73% viability (Fig. 5C). Other basic limonoids, gedunin, azadirone and azadiradione showed cell viability of 50%, 43% and 44% respectively. This was supported by observing the cell morphology by phase contrast microscope (Fig. S3). These results indicate that epoxyazadiradione leads to the formation of less toxic Tau species when compared to other limonoids.

### Epoxyazadiradione activates HSF1

**Figure 6:**
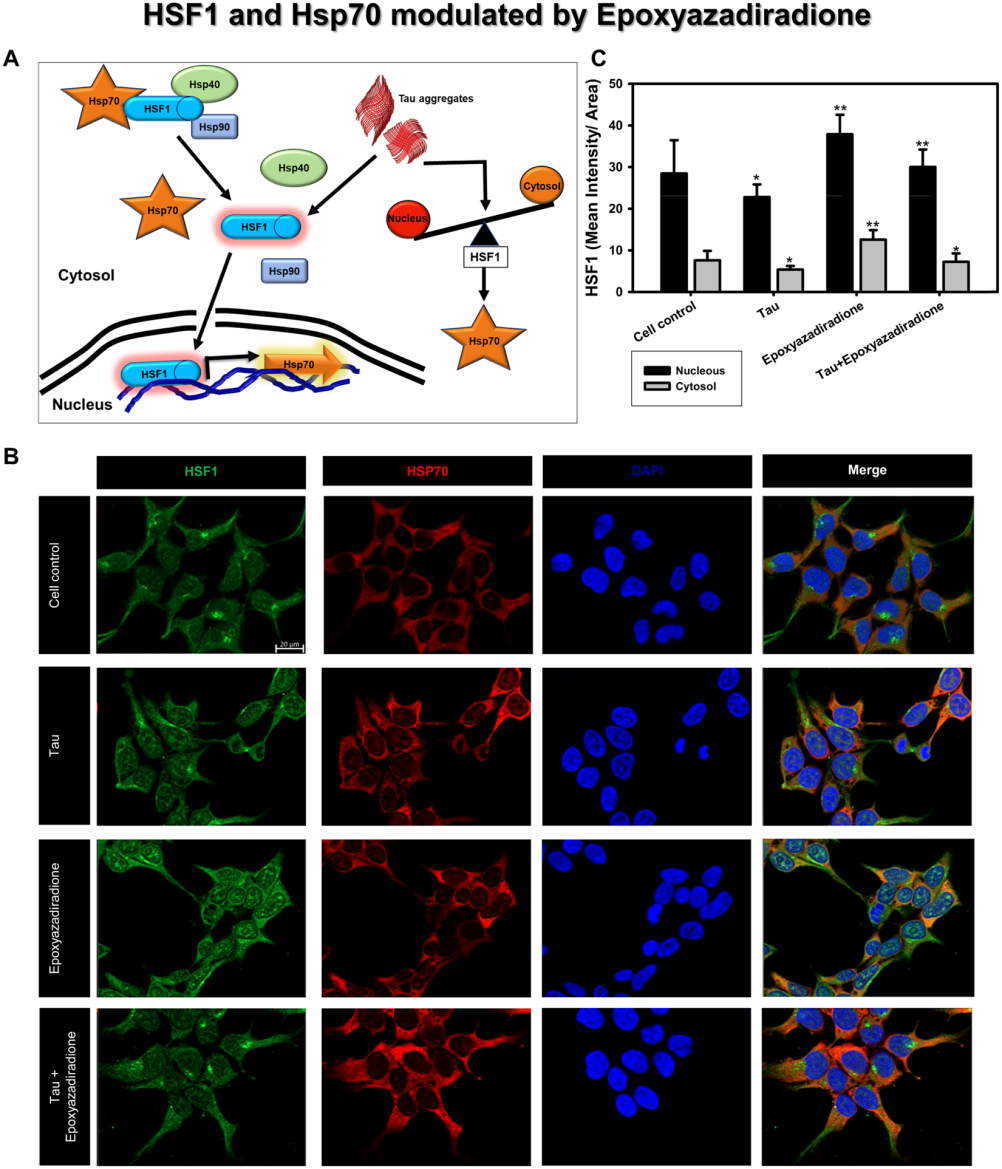
HSF1 localization to nuclear compartment. **(A)** HSF1-Hsp70/40/90 complex are present as inactive form in the cytosol. During stress condition, HSF1 is signalled to dissociate from Hsps and allocated to nucleus for active transcription of Hsp70 in order to manage the cellular protein burden. The altered balance between nuclear *vs*. cytosolic HSF1 is the key determinant of controlling effective proteostasis in AD. (**B)** Tau aggregates induced the Hsp70 level in HEK293T cells while epoxyazadiradione has induced the HSF1 with reduced Hsp70 level, which indicates the feed-back control of cellular proteostasis signalling. (**C)** The quantification showed increase in nuclear as well as cytosolic levels of HSF1 in epoxyazadiradione treatment which is also maintained in Tau stressed cells. Quantification was carried out by Zen 2.3 software (https://www.zeiss.com/microscopy/int/products/light-microscopes/axio-observer-for-biology.html).

**Figure 7:**
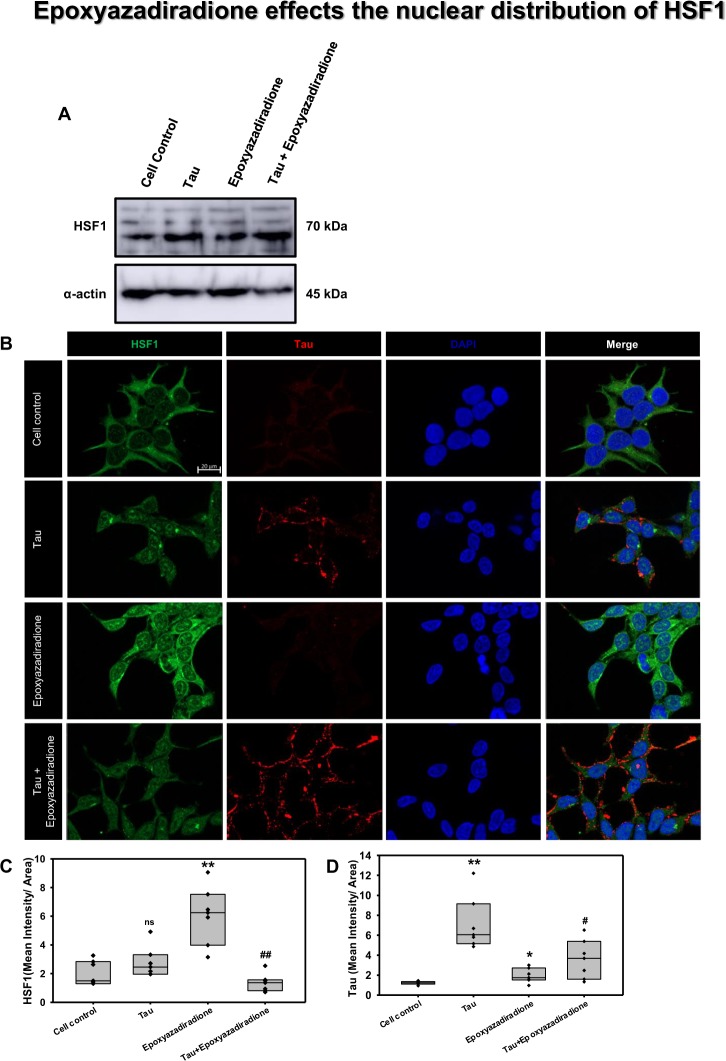
Activation of HSF1 by epoxyazadiradione. (**A)** Treatment of HEK293T cells with Tau and epoxyazadiradione exhibited no changes of HSF1 in western blotting analysis and its quantification. (**B)** Extracellular Tau aggregates reduced the cellular HSF1 level and epoxyazadiradione increased HSF1 level in cytosol and nucleus. Tau aggregates were found to adhere in the cell periphery whereas the intracellular Tau level was negligible in HEK293T cells. (**C,D)** IF quantification showed a significant increase in intracellular HSF1 and Tau aggregates were observed on the cellular periphery. Quantification was carried out by Zen 2.3 software (https://www.zeiss.com/microscopy/int/products/light-microscopes/axio-observer-for-biology.html).

The chaperones are attributed to reduce in AD pathology thus, are unable to clear the aberrant Tau species from the cells. The levels of Hsp70 can be replenished by activating HSF1, a transcription factor for Hsp70. At basal conditions HSF1 is present in cytosol as inactive monomer, its activation is triggered by proteotoxicity, in our study by accumulation of Tau (Fig. 6A)^53^. Thus activated HSF1 enters nucleus and increases the expression of Hsp70. In general the proteotoxicity leads to increase in HSF1 levels but, western blotting suggests that treatment with Tau or epoxyazadiradione did not alter the total HSF1 in HEK293T cells (Fig. 7A). Further we studied the activation of HSF1 and its distribution in nucleus by immunofluorescence microscopy. In our present study, treatment with epoxyazadiradione activated HSF1 and its accumulation in nucleus, compared with Tau treated group as depicted by fluorescence quantification (Fig. 7B,C). The effect was more pronounced in the presence of epoxyazadiradione when compared to cell control and Tau treated groups (Fig. 6B,C). The Tau fibrils were found to adhere onto the cell membrane where epoxyazadiradione unable to degrade the Tau fibrils, which were already, stuck onto the HEK293T cells (Fig. 7B,D). Together, our study suggested that the accumulation of Tau around the cell membrane cannot be cleared by this basic limonoid while the functionalization of epoxyazadiradione depends on the nuclear translocation of HSF1 to instigate the proteostasis machinery for clearance of Tau species.

### Epoxyazadiradione modulate the expression of Hsp70

**Figure 8:**
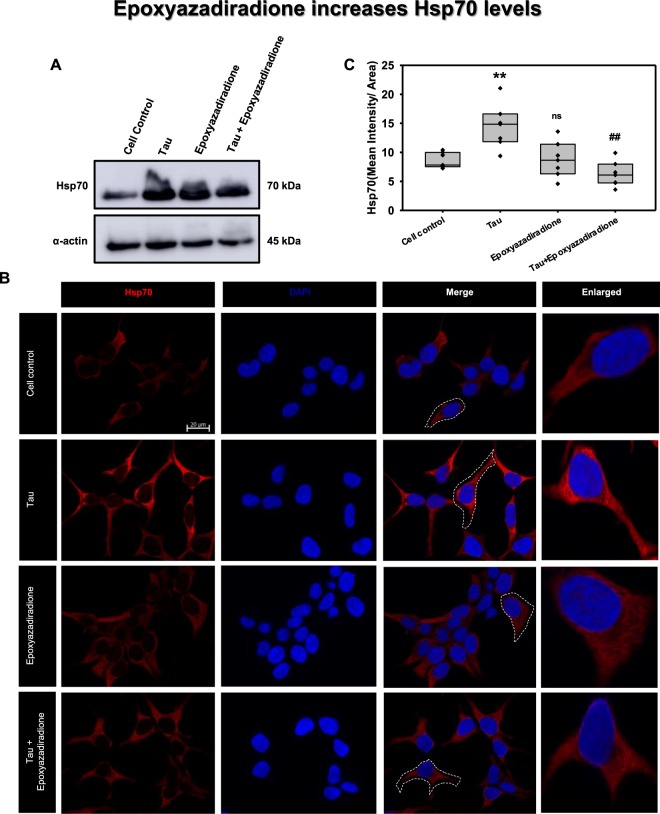
Hsp70 levels aggravated on epoxyazadiradione treatment. (**A**) Hsp70, an indispensable chaperone involved in proteotoxic stress, its levels are increased when cells are exposed to Tau. (**B**) Tau aggregates increased the Hsp70 level in HEK293T cells as compared to cell control where the cells showed an extended morphology. (**C**) Epoxyazadiradione alone and together with Tau aggregates have increased the cellular Hsp70 levels, which indicates the induction of cellular proteostasis machinery in the context of extracellular protein burden. Quantification was carried out by Zen 2.3 software (https://www.zeiss.com/microscopy/int/products/light-microscopes/axio-observer-for-biology.html).

**Figure 9:**
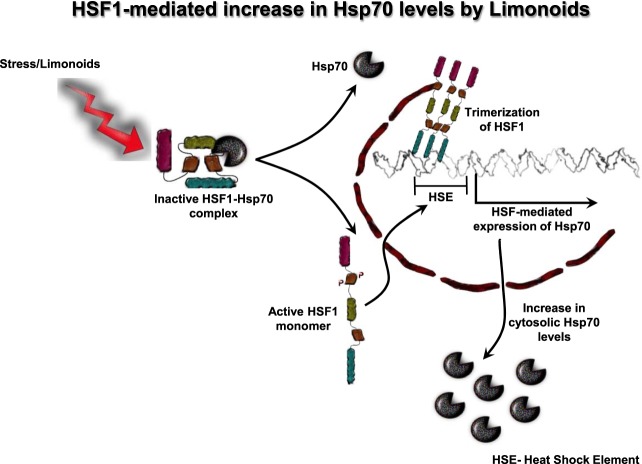
Proposed mechanism for HSF1-mediated clearance of Tau. In normal conditions HSF1 is present as an inactive monomer in cytosol. HSF1 in inactive state forms complex with Hsp70. Triggering of Tau-induced stress or treatment of cells with limonoids leads to dissociation of Hsp70 from HSF1. HSF1 monomer is now subjected to phosphorylation and is transported into nucleus. In nucleus, HSF1 trimerizes and interacts with heat shock element (HSE) present in the upstream of Hsp70 promoter. This leads to increase in expression of Hsp70 and thus enhancing the cytosolic levels of Hsp70.

The AD pathology mitigate the cellular responses, which include diminished levels of Hsp70^54^. Hsp70 abrogates the Tau accumulated in the form of neurofibrillary tangles and aids in preventing neurodegeneration^55,56^. Hence, elevating Hsp70 levels will be a therapeutic approach in overcoming in AD. Elaborating the role of HSF1, the levels of Hsp70 was analysed in presence of Tau and epoxyazadiradione. By western blotting it was observed that Hsp70 level was increased in presence of epoxyazadiradione and Tau treated group (Fig. 8A). Further, a cumulative increase of Hsp70 was observed in epoxyazadiradione and Tau treated group, which may due to the attempt of Hsp70 to eliminate aberrant Tau. Immunofluorescence assay depicted the increase in Hsp70 levels in Tau treatment group when compared to cell control, this was followed by epoxyazadiradione and its presence along with Tau which was correlated with the quantified levels (Fig. 8B,C). This indicate the protective role of epoxyazadiradione in elevating Hsp70 levels in HEK293T cells. This stipulate the protective role of epoxyazadiradione in activating HSF1, which in down-stream might play key role in inducing proteostasis pathways such as chaperone-mediated autophagy, autophagy or ubiquitin-proteasome system (Fig. 9).

## Discussion

The accumulation of mis-folded proteins in intracellular and extracellular milieu of the brain neuronal cells lead to neurodegeneration. AD being the most common form of neurodegenerative disease has gained focus on Tau and its aggregation during pathological conditions. A cascade of events occurs during the cellular dysfunction that triggers the pathological modifications of Tau, leading to its aggregation. Small molecules are being actively screened against these aberrant species in order to overcome AD. Curcumin is a well-known polyphenolic compound isolated from *Curcuma longa*. It was widely studied not only in AD but also in other neurodegenerative diseases such as Parkinson’s disease and was known to inhibit the aggregation of α-synuclein^57,58^. Curcumin was effective in inhibiting the aggregation of α-synuclein, as evidenced by the decrease in fluorescence^59,60^. In the present study, inhibition of Tau aggregation by limonoids also exhibited decrease in fluorescence, as analysed by ThS and ANS binding assay. The inhibitory effect of limonoids are ascertained due to the following properties; azadirone have double bond in the D-ring whereas, azadiradione has an additional carbonyl group, which might contribute for inhibition of Tau aggregation. The epoxidation of double bond on D-ring enhanced the inhibitory activity in epoxyazadiradione. Further, lactonization of D-ring in epoxyazadiradione forms gedunin, exhibit decrease in aggregation inhibition due to increase in volume and rearrangement of the ring. The cyclopentane D-ring along with epoxy and carbonyl group indicates to be a potent pharmacophore in drug development. Basic limonoids such as gedunin and epoxyazadiradione showed potential anti-cancer property^61–64^. The anti-inflammatory role of epoxyazadiradione was elucidated in mice where, it prevented the release of pro-inflammatory cytokine^65^. Azadiradione, an intermediate compound obtained during the biosynthesis of salannin altered the Huntington disease pathology by improving protein quality control system. All these key properties indicate the potential use of limonoids as therapeutics. Apart from these various other cellular functions promote the use of limonoids in AD pathology^59^. Curcumin also targeted Amyloid-β and Tau aggregation thus, assisting in AD prevention^25,66,67^. Yang *et al*., elucidated the role of curcumin in preventing Amyloid-β aggregation as well as solubilizing them *in vitro* and *in vivo* studies^68^. Sedimentation assay showed the distribution of Tau in supernatant and pellet. This states the property of Tau monomers, soluble aggregates or the higher molecular weight aggregates. Monomers and soluble aggregates were separated in supernatant whereas; the higher order aggregates were separated in pellet^69^. The inhibitory role of small molecules were elucidated by presence of protein in supernatant^70^. Screening of different classes of small molecules against Tau aggregation showed that compounds exhibiting inhibition property formed higher molecular weight, SDS-soluble Tau species^69^. Initially it was observed by SDS-PAGE that limonoids accelerated the formation of high molecular weight species, which signifies aggregation of Tau. Since, these higher order aggregates were observed in supernatant, these are suspected to be soluble aggregates, which might be oligomers. At later point of incubation, on pelleting it was observed that Tau was distributed in supernatant and pellet at different ratios. This indicates that limonoids prevented Tau aggregation at variable rates. When compared to other basic limonoids, gedunin exhibited degradation of Tau. In contrast to gedunin, among the other three limonoids, epoxyazadiradione showed higher rates of inhibition (epoxyazadiradione > azadirone > azadiradione).

The TEM analysis of aggregates further distinctly elucidated the inhibitory effect of these limonoids. Resveratrol modulated aggregation of Amyloid-β by accelerating the formation of aggregates from oligomers. However, resveratrol inhibited aggregates formation in monomeric Amyloid-β^17^. The full-length and the repeat Tau monomers formed large aggregates exhibiting varied length and thickness. In presence of basic limonoids, the repeat Tau was aggregated at lower rate. However, the full-length Tau exhibited different morphologies by limonoids. Epoxyazadiradione and azadiradione led to less extensive aggregation of full-length Tau. Azadirone showed the formation of amorphous aggregates. Previously, different studies on Amyloid-β by secondary metabolites such as Oleuropein aglycon and Anthocyanin *etc*., reported similar effects on its aggregation. These amorphous aggregates of Amyloid-β were found to be non-toxic in neuroblastoma, SH-SY5Y and Neuro2a cells^24,71^. Gedunin, unlike other basic limonoids formed extended filaments, which was in accordance with fluorescence analysis. Previously curcumin was also reported to form off-pathway soluble oligomers and aggregates of Amyloid-β that were non-toxic^66^. Hence speculating that the higher order Tau formed by limonoids are not toxic in nature.

Here, we observed that epoxyazadiradione, azadiradione and azadirone showed increase in β-sheet content when compared to Tau alone. Further, the change in ellipticity in presence of azadirone was distinct. This phenomenon explained increased hydrophobicity in Tau upon incubation with Azadirone. The extent of Tau aggregation by gedunin was comparably lesser and the decrease in ellipticity indicated increase in hydrophobicity. Du *et. al*., also elucidated the change in Amyloid-β conformation from random coil to β-sheet structure in presence of brazilin^57,65^. Several other studies also reported similar conformational changes in Amyloid-β^22,24,66,72^. All these observations were associated with the formation of non-toxic protein aggregates. Hence, hypothesizing that these small molecules could act as a sink to trap the aberrant protein and thus overcoming the AD pathology. Similarly, we also accessed the toxicity of these limonoids and also analysed if these compounds were effective in protecting the HEK293T cells from the toxicity of Tau aggregates. Additionally, the increased level of oxidative stress, extracellular plaque deposition by Amyloid-β and activation of apoptotic signalling cascade results in the neuronal death^73,74^. Few acetylcholine esterase (AChE) inhibitors are the current choice of drugs as approved by FDA, although they only ameliorate AD symptoms. Natural compound methoxsalen isolated from *Treculiaobovoidea* (Catterall) and *Angelica archangelica* (Garden Angelica), showed AchE inhibitory activity *in vitro*^75,76^. EGb 761, a formulated leaf extract of *Ginkgo biloba* was extensively used in treatment of all neurodegenerative diseases for memory impairment^13^. Liposome containing curcumin derivatives reduced Amyloid-β induced NMDA (N-acetyl d-Aspartic acid) toxicity in primary astrocytes of Sprague Dawley rats^72,77^. Limonoid, obacunone from the bark of *D. dasycarpus* showed the potent neuroprotective effects on glutamate induced neurotoxicity^78^. Our studies showed that the basic limonoids potentially reduced human Tau-mediated toxicity at a much lower concentration as compared to cytotoxic concentration *i.e*. 50 μM in HEK293T cells. The basic limonoids were not only inhibiting aggregates formation and disintegration of full-length Tau pre-formed aggregates *in vitro*, but also reduced the burden of cellular toxicity by increasing the mitochondrial function as depicted by MTT assay. The HEK293T cells treated with only pre-formed Tau aggregates showed distinct cellular outgrowth at 24 hours of incubation. This was subsequently reversed in a concentration-dependent fashion by addition of basic limonoids. Tau in pathological condition forms different species ranging from oligomers to PHFs. Earlier speculation of PHFs being the toxic species have been disproved on understanding the nucleating effect of oligomeric species^79^. This explains the necessity to identify a potent compound to prevent the formation of toxic oligomeric species^80–82^. In our present study, the toxicity of Tau species formed in presence of basic limonoids was accessed by cell viability assay. Epoxyazadiradione treated Tau resulted in species that are less toxic as examined by the mitochondrial activity. This was followed by gedunin, azadirone and azadiradione. Among the basic limonoids epoxyazadiradione was found to be effective in inhibiting Tau aggregation. It was also essential for small molecules to dissolve pre-formed aggregates and hence the disaggregation property of limonoids would be an advantage in overcoming AD. Earlier studies on disaggregation of pre-formed Amyloid-β aggregates by Tanshinones indicated reduction in fluorescence upon prolonged incubation^83^. Similarly Zhu *et al*., elucidated the role of Baicalein in disaggregating aggregates formed by α-synuclein^71,84^. In our observations, the intensity of fluorescence did not decline, which suggested that limonoids have negligible effect in disintegrating the pre-formed Tau aggregates. However, characterization of Tau by TEM indicated the ability of limonoids to disintegrate preformed aggregates. Further, the cytotoxicity studies also suggested that limonoids aid in rescuing the cell death. Furthermore, the epoxyazadiradione treated Tau aggravated cell viability when compared to Tau; this stimulated to examine the role of epoxyazadiradione in proteostasis pathway. Here, we analysed that epoxyazadiradione stimulated the activity of HSF1 and increased Hsp70 levels. HSF1 plays a key role in proteostasis pathway and known to increase the expression of chaperones during proteostatic stress. HSF1-Hsp70 complex is present in cytosol in inactive form, while in stressed or aging condition; cellular signalling triggers the dissociation of Hsp70 from HSF1, allowing the translocation of HSF1 into nucleus^31,72^. HSF1 in nucleus transcribes the heat shock protein gene locus, which subsequently leads to feed-back inhibition of HSF1 nuclear translocation^85^. HSF1 was known to play crucial role in UPR and heat stress response in rat primary neuronal cells where loss of HSF1 resulted in hyperphosphorylation of Tau^53^. We studied the role of epoxyazadiradione upon subjecting the cells to Tau aggregates stress. Epoxyazadiradione did not alter total HSF1 levels but increased the levels of active HSF1 in nucleus. Previously, azadiradione was known to elevate HSF1 levels in Huntington’s mouse model^59^. Additionally, epoxyazadiradione in the downstream increased Hsp70 levels, which can direct the aberrant Tau towards clearance through chaperone-mediated autophagy, autophagy or ubiquitin-proteasome system. Studying the downstream pathway to address the channelling of Tau towards clearance from the cells would help in gaining the insight towards the role of epoxyazadiradione.

## Conclusion

In present work, we studied the role of basic limonoids isolated from *Azadirachta indica*. The four basic limonoids were screened to analyse their Tau aggregation inhibition potency and disintegration of matured Tau aggregates. Among them epoxyazadiradione was most effective, which would be due to the presence of two oxygen groups in the D-ring. The molecular docking studies also revealed that among the four limonoids, epoxyazadiradione has the highest affinity towards Tau. Further, the MTT assay suggested that limonoids are not toxic to the cells and they reverse the toxicity caused by Tau aggregates at very low concentrations. This elucidates the role of limonoids in preventing Tau aggregation and acting as a molecular sink to trap the pathological Tau into non-toxic conformers. Amongst basic limonoids, role of epoxyazadiradione was elucidated with respect to proteotoxic stress and observed to activate HSF1 that increases cytosolic Hsp70 levels. These results direct the importance of epoxyazadiradione as a therapeutic lead to overcome AD.

## Supporting Information

Supplementary Information.
Supplementary Information 2.
Supplementary Information 3.

## References

[1] Moya K, Benowitz L, Schneider G, Allinquant B (1994). The amyloid precursor protein is developmentally regulated and correlated with synaptogenesis. Developmental Biology.

[2] Zou C (2016). Amyloid precursor protein maintains constitutive and adaptive plasticity of dendritic spines in adult brain by regulating D‐serine homeostasis. The EMBO Journal.

[3] Hiltunen M, van Groen T, Jolkkonen J (2009). Functional roles of amyloid-β protein precursor and amyloid-β peptides: evidence from experimental studies. Journal of Alzheimer’s Disease.

[4] Binder LI, Frankfurter A, Rebhun LI (1985). The distribution of tau in the mammalian central nervous system. The Journal of Cell Biology.

[5] Avila J, Lucas JJ, Perez M, Hernandez F (2004). Role of tau protein in both physiological and pathological conditions. Physiological Reviews.

[6] Li W (2009). Inhibition of tau fibrillization by oleocanthal via reaction with the amino groups of tau. Journal of Neurochemistry.

[7] Peterson DW (2009). Cinnamon extract inhibits tau aggregation associated with Alzheimer’s disease *in vitro*. Journal of Alzheimer’s Disease.

[8] Akoury E (2013). Mechanistic basis of phenothiazine‐driven inhibition of Tau aggregation. Angewandte Chemie International Edition.

[9] Wong HE, Kwon I (2011). Xanthene food dye, as a modulator of Alzheimer’s disease amyloid-beta peptide aggregation and the associated impaired neuronal cell function. PloS One.

[10] Necula M, Chirita CN, Kuret J (2005). Cyanine dye N744 inhibits tau fibrillization by blocking filament extension: implications for the treatment of tauopathic neurodegenerative diseases. Biochemistry.

[11] Ono M (2011). Rhodanine and thiohydantoin derivatives for detecting tau pathology in Alzheimer’s brains. ACS Chemical Neuroscience.

[12] Bulic B (2007). Rhodanine‐based tau aggregation inhibitors in cell models of tauopathy. Angewandte Chemie International Edition.

[13] Bastianetto S, Zheng WH, Quirion R (2000). The Ginkgo biloba extract (EGb 761) protects and rescues hippocampal cells against nitric oxide‐induced toxicity: involvement of its flavonoid constituents and protein kinase C. Journal of Neurochemistry.

[14] Lionta E, Spyrou G, K Vassilatis D, Cournia Z (2014). Structure-based virtual screening for drug discovery: principles, applications and recent advances. Current Topics in Medicinal Chemistry.

[15] Sonawane S, Balmik A, Boral D, Ramasamy S, Chinnathambi S (2019). Baicalein suppresses Tau fibrillization by sequestering oligomers. Archives of Biochemistry and Biophysics.

[16] Mohamed T, Hoang T, Jelokhani-Niaraki M, Rao PP (2013). Tau-derived-hexapeptide 306VQIVYK311 aggregation inhibitors: nitrocatechol moiety as a pharmacophore in drug design. ACS Chemical Neuroscience.

[17] Ladiwala ARA (2010). Resveratrol selectively remodels soluble oligomers and fibrils of amyloid Aβ into off-pathway conformers. Journal of Biological Chemistry.

[18] Chanvitayapongs S, Draczynska-Lusiak B, Sun AY (1997). Amelioration of oxidative stress by antioxidants and resveratrol in PC12 cells. Neuroreport.

[19] Kim Y (2006). Resveratrol inhibits inducible nitric oxide synthase and cyclooxygenase-2 expression in β-amyloid-treated C6 glioma cells. International Journal of Molecular Medicine.

[20] Walker JM (2015). Beneficial effects of dietary EGCG and voluntary exercise on behavior in an Alzheimer’s disease mouse model. Journal of Alzheimer’s Disease.

[21] Rezai-Zadeh K (2005). Green tea epigallocatechin-3-gallate (EGCG) modulates amyloid precursor protein cleavage and reduces cerebral amyloidosis in Alzheimer transgenic mice. Journal of Neuroscience.

[22] Feng Y (2009). Ellagic acid promotes Aβ42 fibrillization and inhibits Aβ42-induced neurotoxicity. Biochemical and Biophysical Research Communications.

[23] Daccache A (2011). Oleuropein and derivatives from olives as Tau aggregation inhibitors. Neurochemistry International.

[24] Rigacci S (2011). Aβ (1-42) aggregates into non-toxic amyloid assemblies in the presence of the natural polyphenol oleuropein aglycon. Current Alzheimer Research.

[25] Abuznait AH, Qosa H, Busnena BA, El Sayed KA, Kaddoumi A (2013). Olive-oil-derived oleocanthal enhances β-amyloid clearance as a potential neuroprotective mechanism against Alzheimer’s disease: *in vitro* and *in vivo* studies. ACS Chemical Neuroscience.

[26] t Hart BA, Copray S, Philippens I (2014). Apocynin, a low molecular oral treatment for neurodegenerative disease. BioMed Research International.

[27] Kiasalari Z (2017). Ellagic acid ameliorates learning and memory deficits in a rat model of Alzheimer’s disease: an exploration of underlying mechanisms. Psychopharmacology.

[28] Sabogal-Guáqueta AM (2015). The flavonoid quercetin ameliorates Alzheimer’s disease pathology and protects cognitive and emotional function in aged triple transgenic Alzheimer’s disease model mice. Neuropharmacology.

[29] Brehme M, Voisine C (2016). Model systems of protein-misfolding diseases reveal chaperone modifiers of proteotoxicity. Disease Models & Mechanisms.

[30] Pierce A (2013). Over‐expression of heat shock factor 1 phenocopies the effect of chronic inhibition of TOR by rapamycin and is sufficient to ameliorate Alzheimer’s‐like deficits in mice modeling the disease. Journal of Neurochemistry.

[31] Neef DW, Jaeger AM, Thiele DJ (2011). Heat shock transcription factor 1 as a therapeutic target in neurodegenerative diseases. Nature Reviews Drug Discovery.

[32] Gomez-Pastor R (2017). Abnormal degradation of the neuronal stress-protective transcription factor HSF1 in Huntington’s disease. Nature Communications.

[33] Roy A, Saraf S (2006). Limonoids: overview of significant bioactive triterpenes distributed in plants kingdom. Biological and Pharmaceutical Bulletin.

[34] Tundis R, Loizzo MR, Menichini F (2014). An overview on chemical aspects and potential health benefits of limonoids and their derivatives. Critical Reviews in Food Science and Nutrition.

[35] Haldar S, Phapale PB, Kolet SP, Thulasiram HV (2013). Expedient preparative isolation, quantification and characterization of limonoids from Neem fruits. *Analytical*. Methods.

[36] Gorantla NV, Shkumatov AV, Chinnathambi S (2017). Conformational Dynamics of Intracellular Tau Protein Revealed by CD and SAXS. Methods in Molecular Biology.

[37] Pence HE, Williams A (2010). ChemSpider: an online chemical information resource. Journal of Chemical Education.

[38] Vandermeersch, T. & Hutchison, G. Open Babel: AN Open Chemical Toolbox. *Journal of Chemoinformatics* **3** (2011). 10.1186/1758-2946-3-33 PMC3198950 21982300

[39] Frisch M. J. *et al*., editors. Gaussian 09, Revision A.02. Gaussian Inc; Wallingford, CT, USA (2009).

[40] Becke AD (1993). Becke’s three parameter hybrid method using the LYP correlation functional. The Journal of Chemical Physics.

[41] Berman HM (2000). The protein data Bank. Nucleic Acids Research.

[42] Zhang W (2019). Heparin-induced tau filaments are polymorphic and differ from those in Alzheimer’s and Pick’s diseases. Elife.

[43] De Lano, W. The PyMOL Molecular Graphics System, Version 1.2 r3pre, Schrödinger, LLC (2002).

[44] Heo L, Park H, Seok C (2013). GalaxyRefine: protein structure refinement driven by side-chain repacking. Nucleic Acids Research.

[45] Lee GR, Heo L, Seok C (2016). Effective protein model structure refinement by loop modeling and overall relaxation. Proteins: Structure, Function, and Bioinformatics.

[46] Sanner MF (1999). Python: a programming language for software integration and development. J. Mol. Graph. Model.

[47] Morris GM (2009). AutoDock4 and AutoDockTools4: Automated docking with selective receptor flexibility. Journal of computational chemistry.

[48] Laskowski RA, Swindells MB (2011). LigPlot+: multiple ligand–protein interaction diagrams for drug discovery. Journal of Chemical Information and Modeling.

[49] Wallace AC, Laskowski RA, Thornton JM (1995). LIGPLOT: a program to generate schematic diagrams of protein-ligand interactions. Protein Engineering, Design and Selection.

[50] Santa-María I, Pérez M, Hernández F, Avila J, Moreno FJ (2006). Characteristics of the binding of thioflavin S to tau paired helical filaments. Journal of Alzheimer’s Disease.

[51] Gorantla NV, Khandelwal P, Poddar P, Chinnathambi S (2017). Global Conformation of Tau Protein Mapped by Raman Spectroscopy. Methods in Molecular Biology.

[52] Serrano-Pozo A, Frosch MP, Masliah E, Hyman BT (2011). Neuropathological alterations in Alzheimer disease. Cold Spring Harbor Perspectives in Medicine.

[53] Kim E, Sakata K, Liao F-F (2017). Bidirectional interplay of HSF1 degradation and UPR activation promotes tau hyperphosphorylation. Plos Genetics.

[54] Repalli J, Meruelo D (2015). Screening strategies to identify HSP70 modulators to treat Alzheimer’s disease. Drug Design, Development and Therapy.

[55] Patterson KR (2011). Heat shock protein 70 prevents both tau aggregation and the inhibitory effects of preexisting tau aggregates on fast axonal transport. Biochemistry.

[56] Voss K, Combs B, Patterson KR, Binder LI, Gamblin TC (2012). Hsp70 alters tau function and aggregation in an isoform specific manner. Biochemistry.

[57] Du W-J (2015). Brazilin inhibits amyloid β-protein fibrillogenesis, remodels amyloid fibrils and reduces amyloid cytotoxicity. Scientific Reports.

[58] Ji H-F, Shen L (2014). The multiple pharmaceutical potential of curcumin in Parkinson’s disease. CNS & Neurological Disorders-Drug Targets (Formerly Current Drug Targets-CNS & Neurological Disorders).

[59] Singh BK (2018). Azadiradione restores protein quality control and ameliorates the disease pathogenesis in a mouse model of Huntington’s disease. Molecular Neurobiology.

[60] Singh PK (2012). Curcumin modulates α-synuclein aggregation and toxicity. ACS Chemical Neuroscience.

[61] Kamath SG (2009). Gedunin, a novel natural substance, inhibits ovarian cancer cell proliferation. International Journal of Gynecologic Cancer.

[62] Kikuchi T (2011). Cytotoxic and apoptosis-inducing activities of limonoids from the seeds of Azadirachta indica (neem). Journal of Natural Products.

[63] Kumar D (2018). Epoxyazadiradione suppresses breast tumor growth through mitochondrial depolarization and caspase-dependent apoptosis by targeting PI3K/Akt pathway. BMC Cancer.

[64] Shilpa G (2017). Epoxyazadiradione purified from the Azadirachta indica seed induced mitochondrial apoptosis and inhibition of NFκB nuclear translocation in human cervical cancer cells. Phytotherapy Research.

[65] Alam A (2012). Novel anti-inflammatory activity of epoxyazadiradione against macrophage migration inhibitory factor inhibition of tautomerase and proinflammatory activities of macrophage migration inhibitory factor. Journal of Biological Chemistry.

[66] Thapa A, Jett SD, Chi EY (2015). Curcumin attenuates amyloid-β aggregate toxicity and modulates amyloid-β aggregation pathway. ACS Chemical Neuroscience.

[67] Ma Q-L (2013). Curcumin suppresses soluble tau dimers and corrects molecular chaperone, synaptic, and behavioral deficits in aged human tau transgenic mice. Journal of Biological Chemistry.

[68] Yang F (2005). Curcumin inhibits formation of amyloid β oligomers and fibrils, binds plaques, and reduces amyloid *in vivo*. Journal of Biological Chemistry.

[69] Taniguchi S (2005). Inhibition of heparin-induced tau filament formation by phenothiazines, polyphenols, and porphyrins. Journal of Biological Chemistry.

[70] Masuda M (2006). Small molecule inhibitors of α-synuclein filament assembly. Biochemistry.

[71] Yamakawa MY (2016). Anthocyanin suppresses the toxicity of Aβ deposits through diversion of molecular forms in *in vitro* and *in vivo* models of Alzheimer’s disease. Nutritional Neuroscience.

[72] Demirovic D, de Toda IM, Nizard C, Rattan SI (2014). Differential translocation of heat shock factor-1 after mild and severe stress to human skin fibroblasts undergoing aging *in vitro*. Journal of Cell Communication and Signaling.

[73] Pugazhenthi S, Wang M, Pham S, Sze C-I, Eckman CB (2011). Downregulation of CREB expression in Alzheimer’s brain and in Aβ-treated rat hippocampal neurons. Molecular Neurodegeneration.

[74] Oguchi T (2017). Cilostazol suppresses Aβ-induced neurotoxicity in SH-SY5Y cells through inhibition of oxidative stress and MAPK signaling pathway. Frontiers in Aging Neuroscience.

[75] Sigurdsson S, Gudbjarnason S (2007). Inhibition of acetylcholinesterase by extracts and constituents from Angelica archangelica and Geranium sylvaticum. Zeitschrift für Naturforschung C.

[76] Kim, J. K. *et al*. Inhibitory effect of Poncirus trifoliate on acetylcholinesterase and attenuating activity against trimethyltin-induced learning and memory impairment. *Bioscience, Biotechnology, and Biochemistry*, 0904061398–0904061398 (2009). 10.1271/bbb.80859 19420715

[77] Lin M-S (2011). Curcumin enhances neuronal survival in N-methyl-d-aspartic acid toxicity by inducing RANTES expression in astrocytes via PI-3K and MAPK signaling pathways. Progress in Neuro-Psychopharmacology and Biological Psychiatry.

[78] Jeong G-S (2010). Neuroprotective effects of constituents of the root bark of Dictamnus dasycarpus in mouse hippocampal cells. Archives of Pharmacal Research.

[79] Flach K (2012). Tau oligomers impair artificial membrane integrity and cellular viability. Journal of Biological Chemistry.

[80] Pickhardt M, Lawatscheck C, Börner HG, Mandelkow E (2017). Inhibition of Tau protein aggregation by rhodanine-based compounds solubilized via specific formulation additives to improve bioavailability and cell viability. Current Alzheimer Research.

[81] Paranjape SR (2015). Azaphilones inhibit tau aggregation and dissolve tau aggregates *in vitro*. ACS Chemical Neuroscience.

[82] Pickhardt M (2007). Phenylthiazolyl-hydrazide and its derivatives are potent inhibitors of τ aggregation and toxicity *in vitro* and in cells. Biochemistry.

[83] Wang Q (2013). Tanshinones inhibit amyloid aggregation by amyloid-β peptide, disaggregate amyloid fibrils, and protect cultured cells. ACS Chemical Neuroscience.

[84] Zhu M (2004). The flavonoid baicalein inhibits fibrillation of α-synuclein and disaggregates existing fibrils. Journal of Biological Chemistry.

[85] Lackie RE (2017). The Hsp70/Hsp90 chaperone machinery in neurodegenerative diseases. Frontiers in Neuroscience.

