# Supplementary material for: Basic Limonoid modulates Chaperone-mediated Proteostasis and dissolve Tau fibrils
Source: Sci Rep. 2020 Mar 4;10:4023. doi: 10.1038/s41598-020-60773-1 (PMC7055235; doi:10.1038/s41598-020-60773-1)
Supplement: Supplementary file 1 — Supplementary Information. [file 41598_2020_60773_MOESM1_ESM.pdf]

SUPPORTING INFORMATION

Basic Limonoid modulates Chaperone-mediated proteostasis and dissolve Tau fibrils

Nalini V. Gorantla,[a],[c] Rashmi Das,[a],[c] Hariharakrishnan Chidambaram,[a],[c] Tushar Dubey,[a],[c Fayaj A. Mulani,[b],[c]] Hirekodathakallu V. Thulasiram,*,[b],[c] and Subashchandrabose Chinnathambi,[a],[c]**

[a]Neurobiology Group, Division of Biochemical Sciences, CSIR-National Chemical Laboratory, Dr. Homi Bhabha Road, 411008 Pune, India.

[b]Division of Organic Chemistry, CSIR-National Chemical Laboratory, Dr. Homi Bhabha Road, 411008 Pune, India.

[c]Academy of Scientific and Innovative Research (AcSIR), 411008 Pune, India.

[§]To whom correspondence should be addressed**:** Total extraction and isolation of Limonoids: **Prof. Hirekodathakallu V. Thulasiram**. Email: hv.thulasiram@ncl.res.in **Prof. Subashchandrabose Chinnathambi**, Neurobiology group, Division of Biochemical Sciences, CSIR-National Chemical Laboratory (CSIR-NCL), Dr. Homi Bhabha Road, 411008 Pune, India, Telephone: +91-20-25902232, Fax. +91-20-25902648. Email: s.chinnathambi@ncl.res.in

Supplementary Methods

Plant material and chemicals

Neem fruits were collected from Aurangabad, Maharashtra (India). HPLC grade solvents were procured from Sigma. LC-MS grade solvents were purchased from JT Baker.

Extraction of neem fruit coat

Neem fruit coat separated from the kernel (1 Kg) was crushed and homogenised using blender. The homogenised tissue was subjected to repeated methanol extraction for three times over a period of 3 hours (2.5 L X 3). The pooled methanol extract was concentrated under reduced pressure and then partitioned between ethylacetate and water. Organic layer obtained was concentrated to obtain crude extract (24.4 g).

Isolation of limonoids by MPLC

One gram of above crude extract was subjected to medium pressure liquid chromatography fitted with 24 g of Redisep pre-packed cartridge. The elution solvent used at flow rate of 20 mL/min and monitored by UV detector at 200-400 nm range as described earlier (1). In short, the following gradient solvent system was used. 5% EtOAc : Petroleum ether; 0.0-3.0 min., 10% EtOAc : Petroleum ether; 6.0 min., 3 min. hold, 15% EtOAc : Petroleum ether; 12.0 min., 3 min. hold, 20% EtOAc : Petroleum ether; 18.0 min., 3 min. hold, 25% EtOAc : Petroleum ether; 24.0 min., 3 min. hold, 30% EtOAc : Petroleum ether; 30.0 min., 6 min. hold, 35% EtOAc : Petroleum ether; 39.0 min., 6 min. hold, 40% EtOAc : Petroleum ether; 48.0 min., 6 min. hold, was used. The purified compounds were identified as azadirone, epoxyazadiradione, azadiradione and gedunin based on various spectral data such as HRPM, MS/MS and NMR with that of reported (1). From one gram of crude extract, 0.04 g of azadirone, 0.21 g epoxyazadiradione, 0.26 g azadiradione and 0.02 g of gedunin were obtained.

LC-(ESI)-HRMS condition

The purity of the isolated basic limonoids such as azadirone, azadiradione, epoxyazadiradione and gedunin, were further confirmed by subjecting to ESI-Orbitrap MS associated with Accela 1250 pump and Accela open AS (Thermoscientific Q Exactive^{TM}; Fig. S6-9). 5.0 µL of the purified limonoids dissolved in methanol (at concentration of 0.1 mg/mL MeOH) was injected on to a Waters Acquity BEH C18 column (1.7 µm, 2.1 mm X 100 mm) with gradient solvent system of methanol : water (0.1% formic acid) with a flow rate of 300 µL/min. The samples were analysed in positive ionisation mode in the scan range of *m/z* 100 to 1000. Limonoids isolated were found to be over 99.5% pure (Figure. S5, S6, S7 and S8).

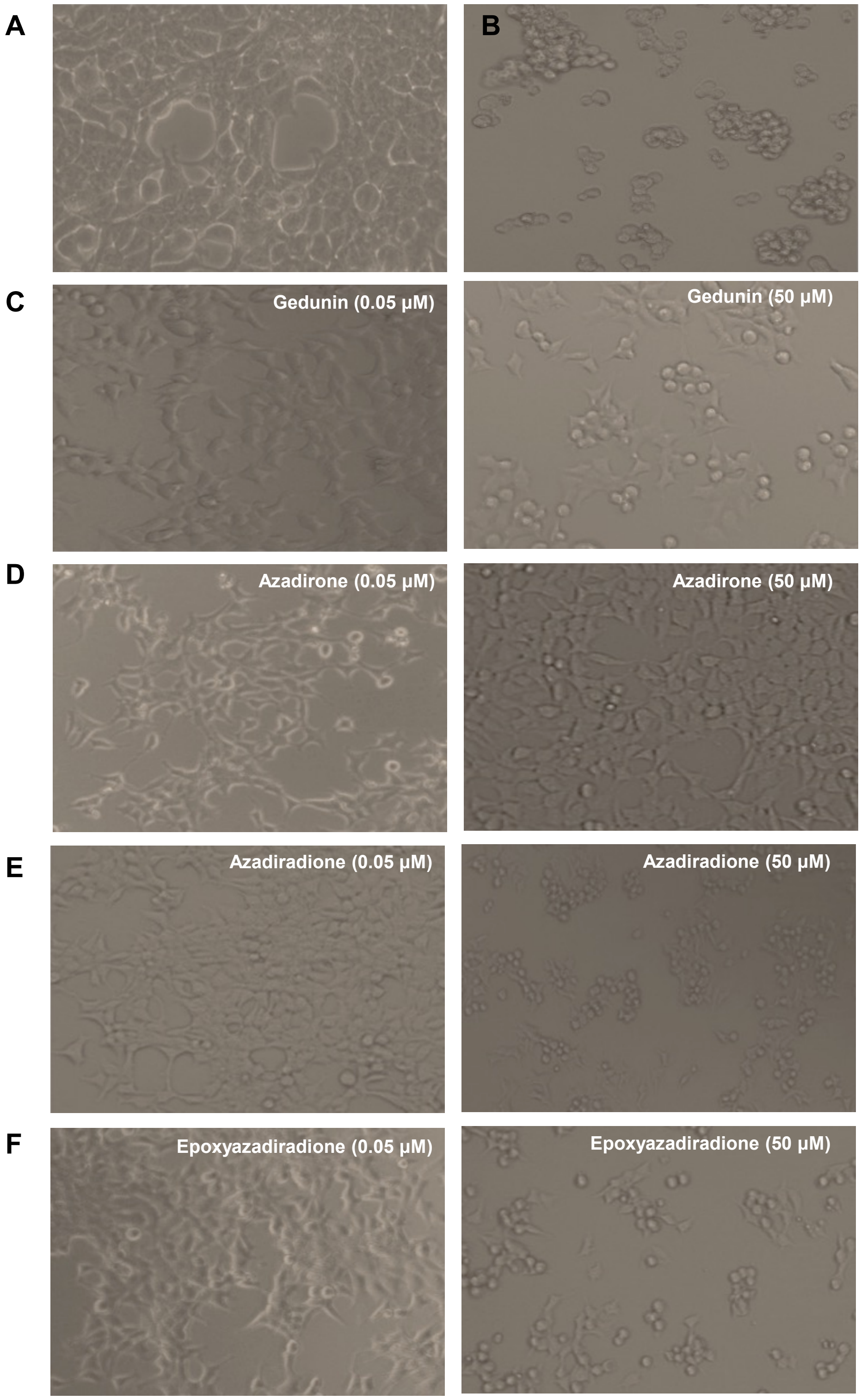

Supplementary 1. Cytotoxicity of basic limonoids on HEK293T cells. 10^4 cells/well were seeded in 96 well plate and treated with 50 µM concentration of basic limonoids (gedunin, azadirone, azadiradione and epoxyazadiradione) for 24 hours at 37°C, 5% CO_2 and the morphological changes were observed under phase contrast microscope at 20X magnification. At 50 µM concentration azadiradione (E) and epoxyazadiradione (F) showed maximum rounding up of the cells than gedunin (C) and azadirone (D) (no rounding up of cells) as compared to negative (5% DMSO) (B) and positive control (A).

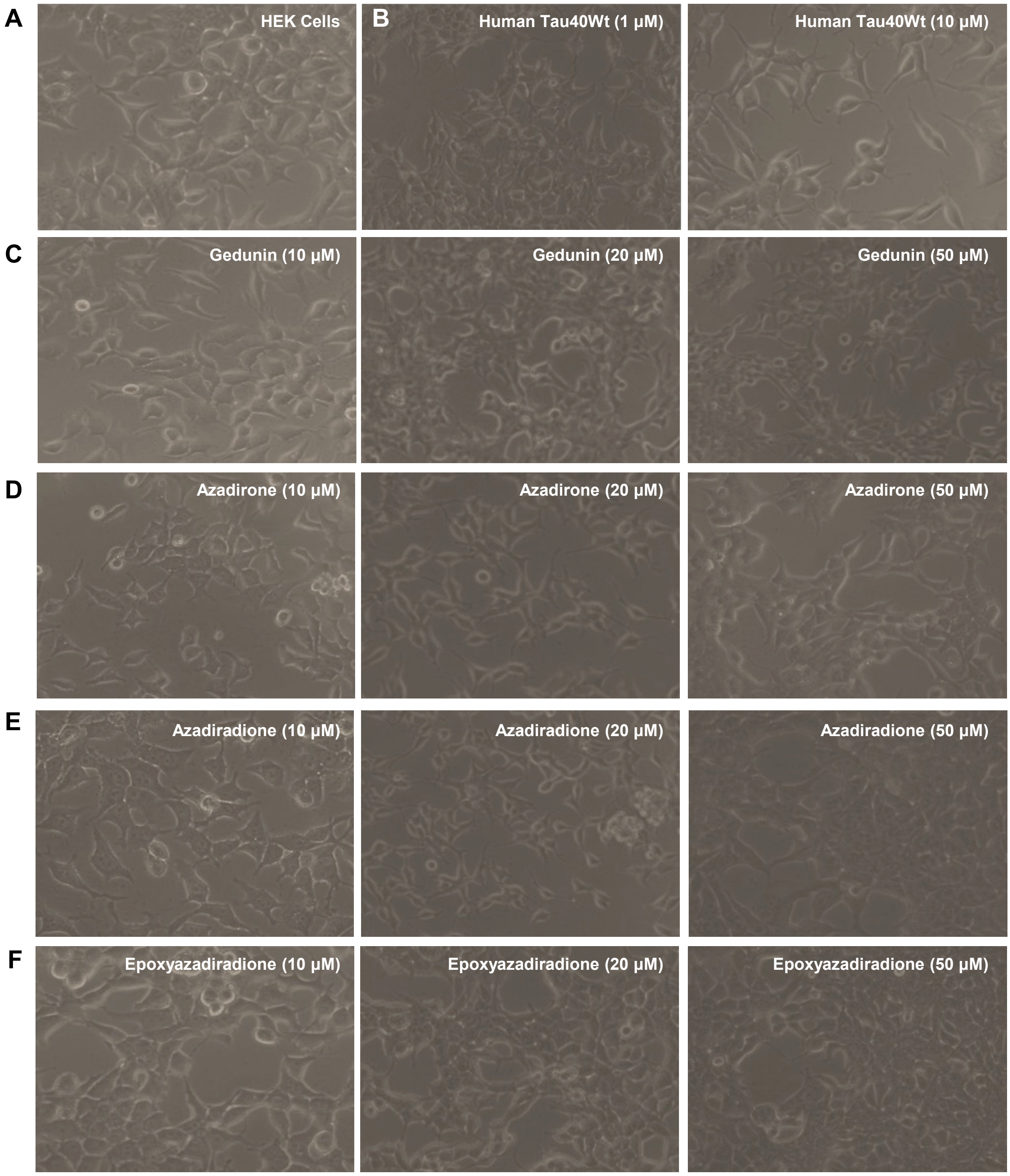

Supplementary 2. Inhibition of full-length Tau aggregates mediated cytotoxicity by basic limonoids on HEK293T cells. 10^4 cells/well were seeded in 96 well plate and co-treated with 10 µM concentration of Tau aggregates and three different concentrations (10, 20 and 50 µM) of gedunin (C), azadirone (D), azadiradione (E), and epoxyazadiradione (F) separately for 24 hours at 37°C, 5% CO_2. After incubation, the cell morphology was observed under phase contrast microscope. A distinct cellular outgrowth or extensions were observed in Tau aggregates treated groups (B) and then the morphology was altered back to the normal (A) on treating with basic limonoids in concentration dependent manner.

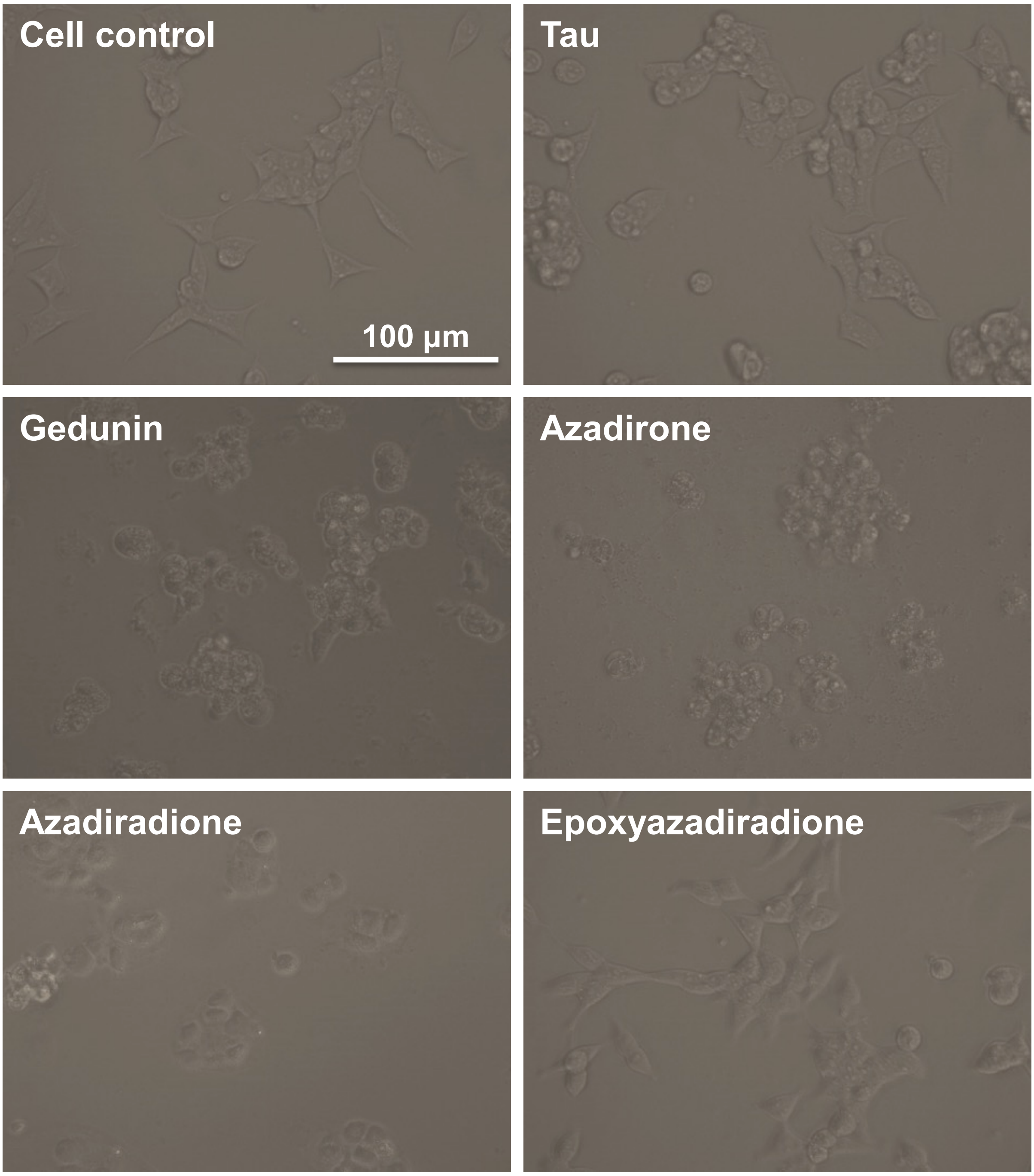

Supplementary 3. Phase contrast images for cell morphology of HEK293T. The cell control showed the native morphology of HEK293T cells. Treatment with Tau showed mixed population of cells with native morphology as well as cells with rounded shape, indicating partial toxicity. Tau species obtained in presence of gedunin, azadirone and azadiradione resulted in shrunken cells with rounded morphology which explains their toxicity. On contrary epoxyazadiradione formed Tau species were non-toxic to cells as the treatment did not change the cell morphology and exhibited cell morphology as in control.

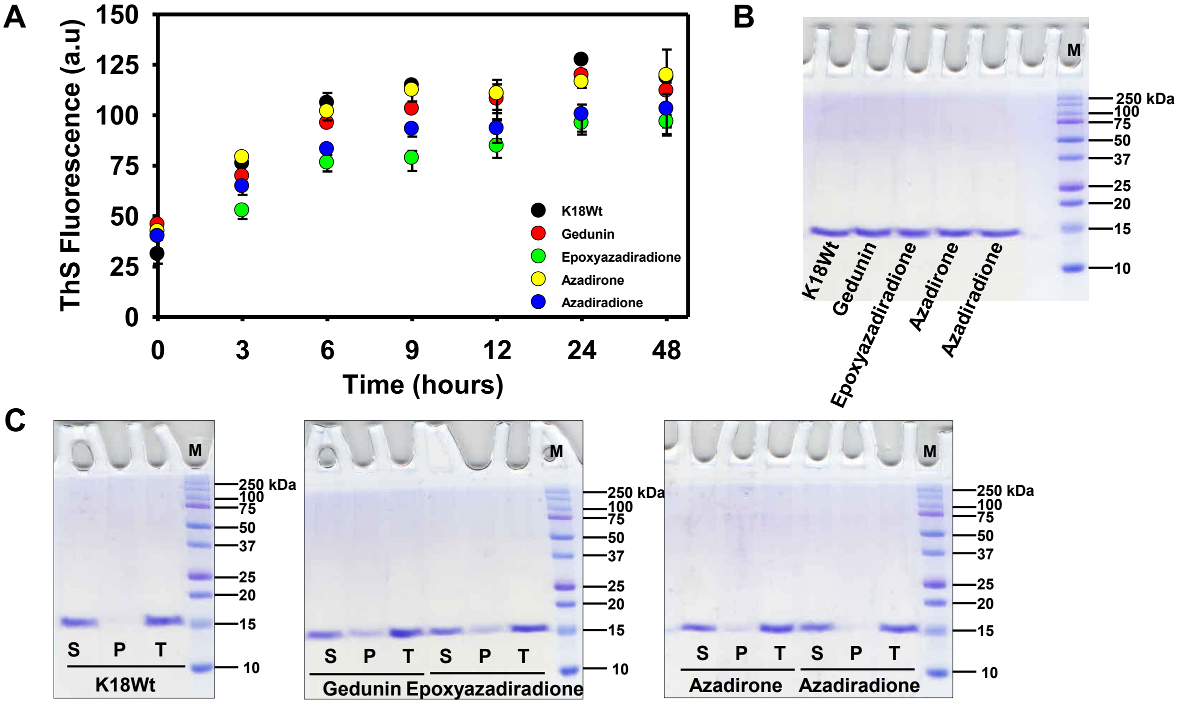

Supplementary 4. The aggregation propensity of Tau inhibition by basic limonoids. A. The ThS fluorescence analysis revealed that epoxyazadiradione had more inhibitory effect on repeat Tau aggregation followed by azadiradione. Gedunin and azadirone exhibited negligible effect in inhibiting repeat Tau aggregation. **B.** The SDS-PAGE analysis of repeat Tau at 0 hour indicated absence of higher order aggregates in presence or absence of limonoids. **C.** However upon sedimentation, the repeat Tau in absence of limonoids was devoid of Tau in pellet (P) fraction and it was completely separated in supernatant (S). This was comparable with the total (T) amount of protein, not subjected to sedimentation. Gedunin, epoxyazadiradione and azadirone led to the separation of Tau into pellet in lower amounts. In presence of azadiradione Tau protein was not obtained in pellet fraction. This indicates that gedunin, epoxyazadiradione and azadirone led to the formation of higher molecular weight Tau that was separated in pellet.

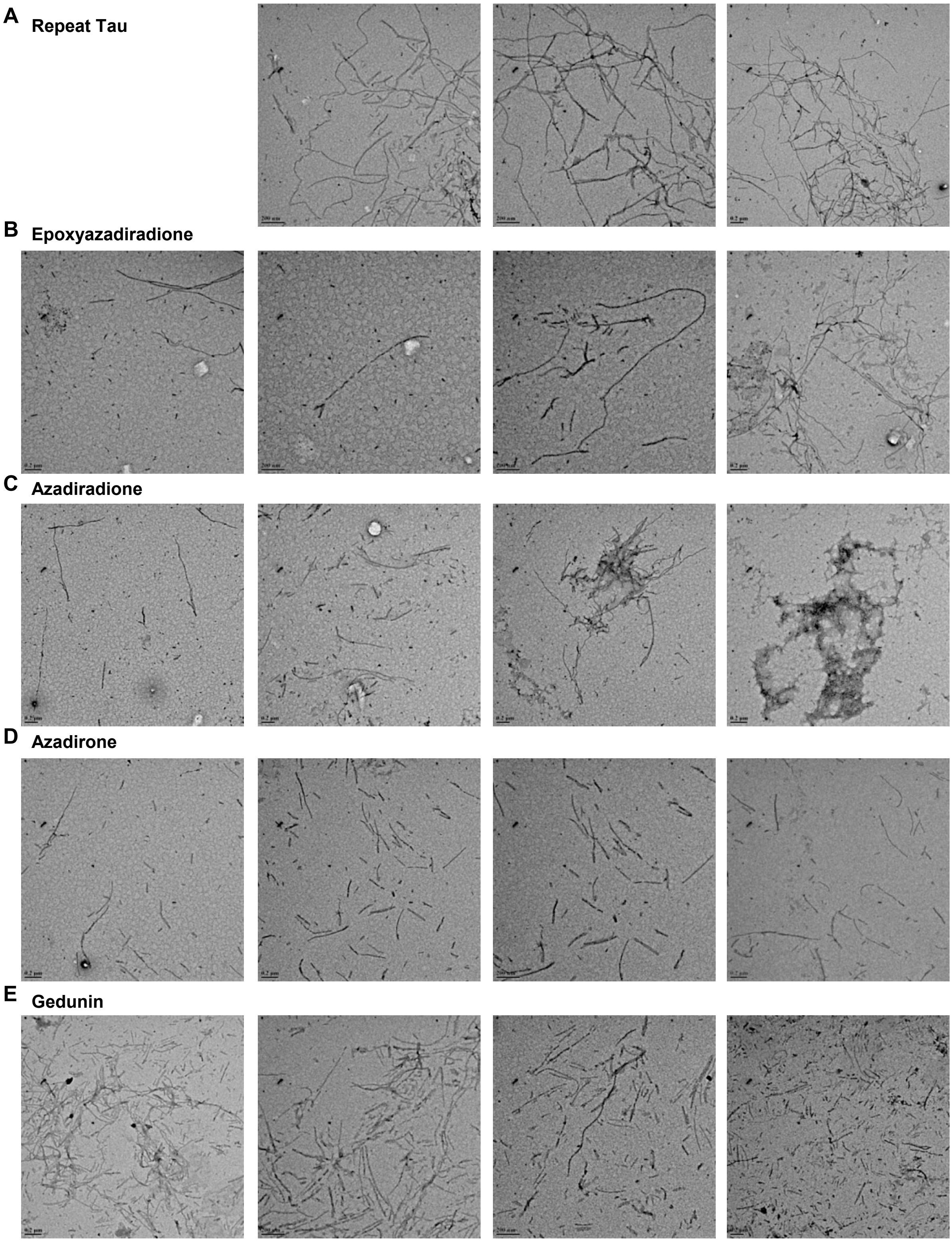

Supplementary 5. Role of basic limonoids in preventing repeat Tau aggregation: A. The repeat Tau aggregated to form long filament like morphology of varied lengths. **B, C, D.** Epoxyazadiradione, azadiradione and azadirone prevented aggregation of Tau and led to the formation of aggregates with reduced length. Azadiradione also exhibited the formation of amorphous aggregates. **E.** Gedunin however, showed mixed morphology of aggregates which resembled like both, control and treated.

A

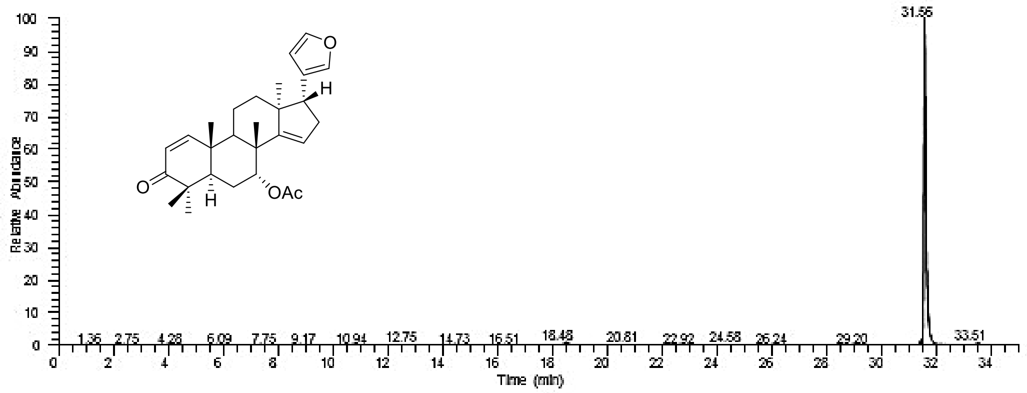

B

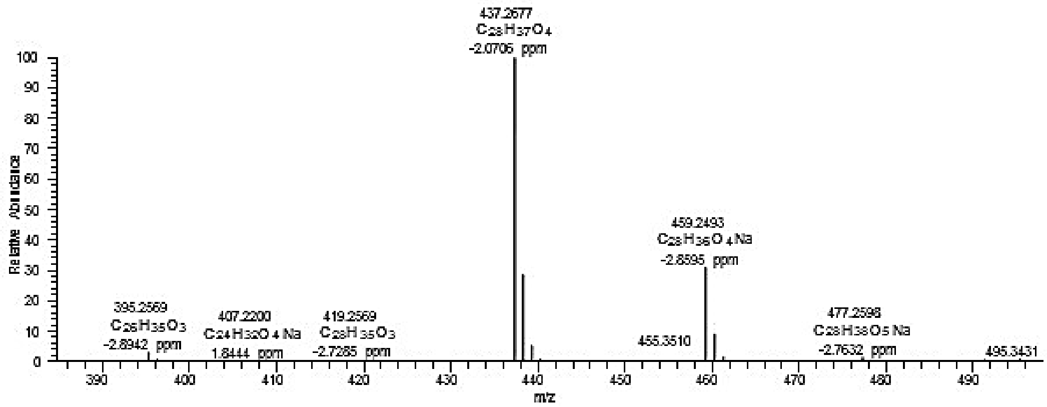

Supplementary 6. A. LC-ESI (+)-HRMS Chromatogram. **B.** Mass spectra of azadirone.

A

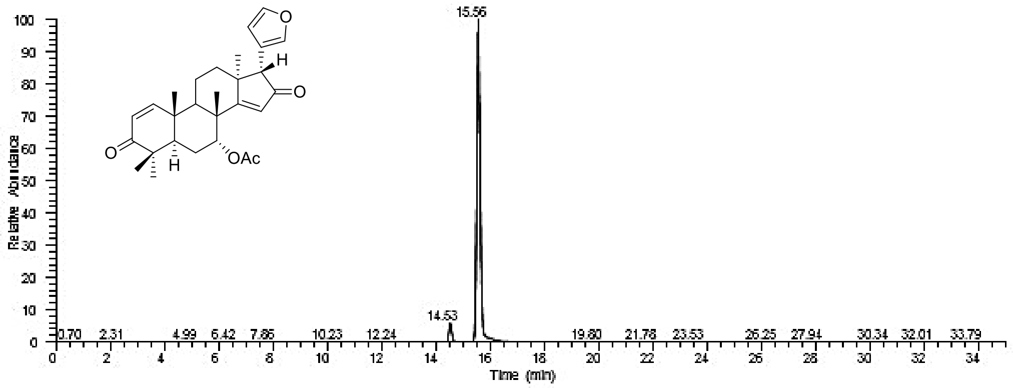

B

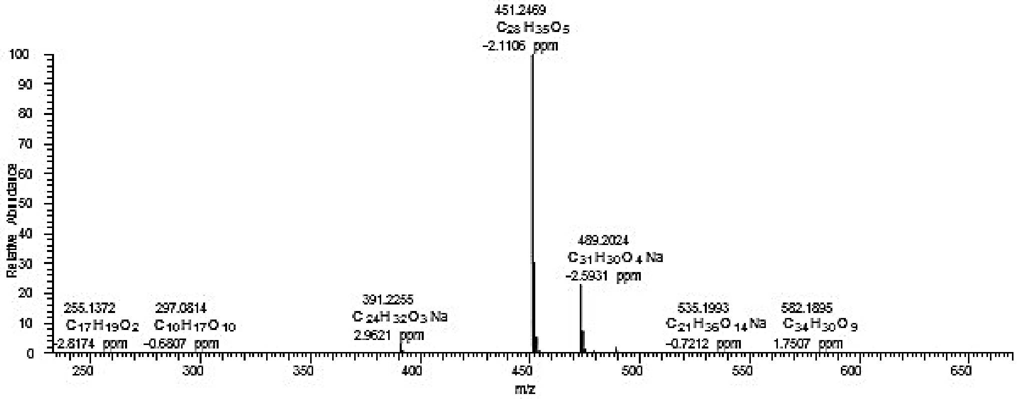

Supplementary 7. A. LC-ESI (+)-HRMS Chromatogram. **B.** Mass spectra of azadiradione.

A

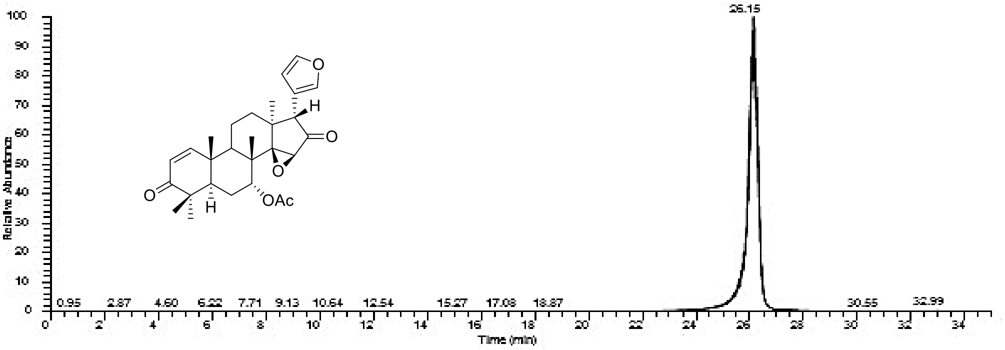

B

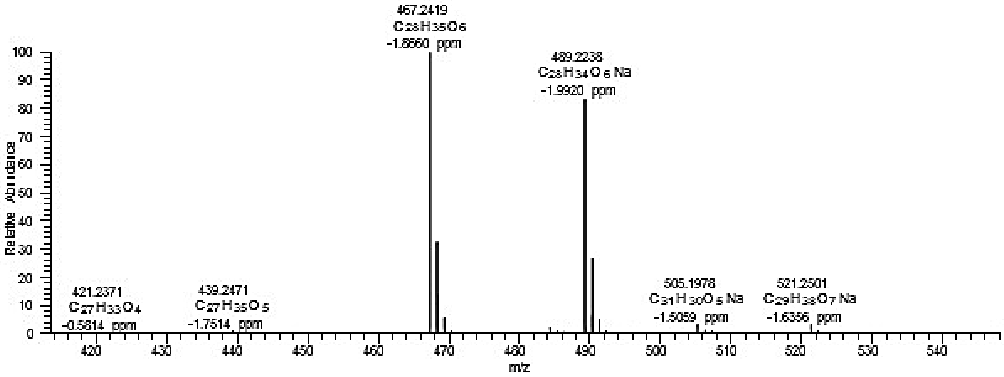

Supplementary 8. A. LC-ESI (+)-HRMS Chromatogram. **B.** Mass spectra of epoxyazadiradione.

A

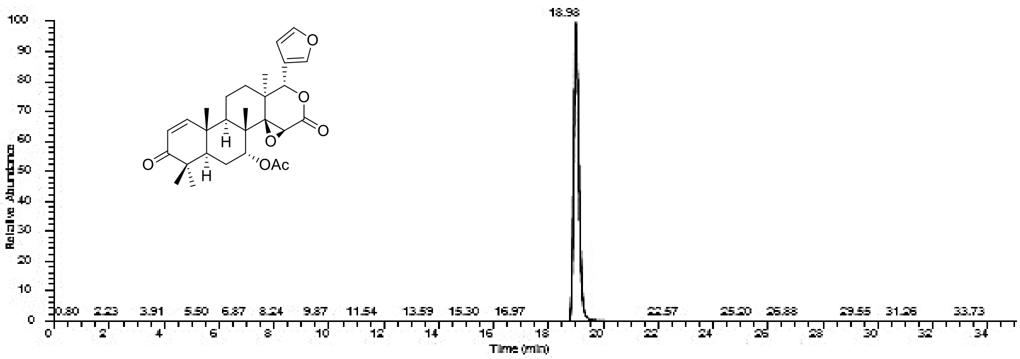

B

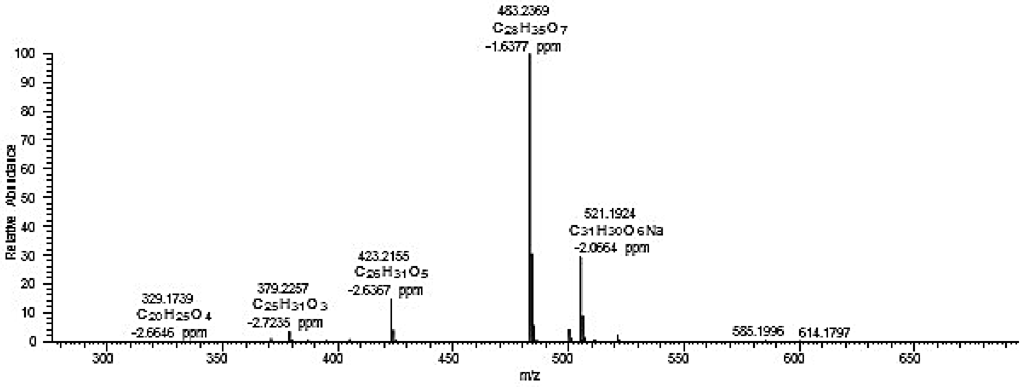

Supplementary 9. A. LC-ESI (+)-HRMS Chromatogram. **B.** Mass spectra of gedunin.

A Azadirone.xyz

68	C -3.642000 -2.370000 -0.782000	H 3.306000 0.773000 1.186000	H 3.717000 -2.084000 -1.045000
00000001	C 0.424000 -1.939000 0.738000	H 1.356000 2.468000 -1.389000	H 0.250000 -1.584000 -2.146000
C 1.305000 0.425000 -0.680000	O -0.841000 2.072000 0.501000	H 3.753000 2.521000 -0.386000	H -0.265000 -0.081000 -2.913000
C 2.377000 -0.590000 -0.210000	C -0.478000 3.402000 0.389000	H 3.907000 1.394000 -1.749000	H -1.454000 -1.131000 -2.097000
C -0.176000 0.049000 -0.740000	O 7.077000 -0.780000 -0.257000	H -0.813000 1.963000 -1.597000	H -1.563000 -2.356000 2.484000
C -2.081000 -1.092000 0.751000	C 5.956000 -0.036000 -0.653000	H -1.796000 -3.109000 -0.120000	H -3.223000 -1.776000 2.522000
C -0.542000 -0.738000 0.560000	C 5.427000 -1.118000 1.250000	H -3.098000 1.912000 -0.792000	H -1.894000 -0.659000 2.884000
C -3.016000 0.148000 0.461000	C 6.709000 -1.435000 0.923000	H -2.799000 0.435000 -1.701000	H -4.926000 -1.806000 1.766000
C 1.872000 1.589000 -1.026000	O -5.632000 -1.354000 -1.592000	H 1.849000 -0.795000 1.885000	H -6.317000 -0.800000 1.360000
C 3.377000 1.582000 -0.805000	O -0.287000 3.970000 -0.683000	H 2.541000 -2.267000 1.169000	H -5.033000 -0.142000 2.402000
C 3.556000 0.393000 0.186000	C 2.834000 -1.520000 -1.362000	H -3.864000 -3.242000 -1.388000	H -5.072000 1.389000 -1.152000
C -1.067000 1.303000 -0.765000	C -0.436000 -0.742000 -2.056000	H 0.407000 -2.583000 -0.147000	H -5.285000 1.811000 0.569000
C -4.536000 -0.220000 0.262000	C -2.206000 -1.503000 2.258000	H 0.127000 -2.555000 1.593000	H -6.419000 0.710000 -0.255000
C -2.494000 -2.276000 -0.095000	C -5.237000 -0.785000 1.538000	H 6.075000 0.543000 -1.550000	H 0.490000 3.503000 2.291000
C -2.554000 0.966000 -0.776000	C -5.372000 1.010000 -0.173000	H 4.880000 -1.464000 2.110000	H -1.249000 3.815000 2.347000
C 1.862000 -1.424000 0.984000	C -0.338000 3.997000 1.772000	H 7.462000 -2.055000 1.372000	H -0.141000 5.066000 1.691000
C -4.674000 -1.314000 -0.804000	H -2.937000 0.816000 1.327000	H 3.094000 -0.936000 -2.250000	
C 4.928000 -0.211000 0.228000	H -0.326000 -0.029000 1.369000	H 2.051000 -2.231000 -1.636000	

B Azadiradione.xyz

67	C -4.084000 -1.858000 -0.166000	H 0.027000 -0.263000 1.105000	H -0.334000 -2.459000 -2.080000
00000001	C 0.156000 -2.374000 0.761000	H 4.471000 -1.720000 -0.657000	H -0.571000 -1.023000 -3.080000
C 1.379000 -0.578000 -1.154000	O -0.019000 1.771000 -0.185000	H 1.793000 1.180000 -2.366000	H -1.846000 -1.558000 -1.952000
C 2.249000 -1.708000 -0.550000	C 0.493000 2.968000 -0.688000	H -0.425000 1.258000 -2.178000	H -1.543000 -1.923000 2.768000
C -0.137000 -0.558000 -0.994000	O 4.535000 0.551000 -2.209000	H -2.406000 -2.983000 0.396000	H -2.994000 -0.935000 2.875000
C -2.013000 -0.861000 0.892000	O 4.899000 0.520000 2.686000	H -2.460000 2.009000 -1.108000	H -1.400000 -0.157000 2.869000
C -0.486000 -0.993000 0.467000	C 5.001000 -0.361000 1.600000	H -2.699000 0.373000 -1.709000	H -4.731000 -0.535000 2.364000
C -2.622000 0.535000 0.473000	C 3.048000 0.754000 1.402000	H 1.961000 -1.532000 1.591000	H -5.854000 0.750000 1.924000
C 2.137000 0.317000 -1.814000	C 3.678000 1.186000 2.528000	H 2.164000 -3.194000 1.024000	H -4.285000 1.168000 2.651000
C 3.579000 0.032000 -1.633000	O -5.857000 -0.459000 -0.901000	H -4.625000 -2.713000 -0.558000	H -4.523000 2.040000 -1.112000
C 3.654000 -1.016000 -0.480000	O 0.518000 3.233000 -1.885000	H -0.143000 -3.110000 0.006000	H -4.319000 2.781000 0.499000
C -0.676000 0.867000 -1.191000	C 2.366000 -2.885000 -1.563000	H -0.169000 -2.759000 1.731000	H -5.826000 1.937000 0.057000
C -4.195000 0.598000 0.530000	C -0.769000 -1.460000 -2.095000	H 5.894000 -0.957000 1.557000	H 0.380000 3.728000 1.315000
C -2.851000 -1.992000 0.343000	C -1.994000 -0.979000 2.454000	H 2.096000 1.084000 1.021000	H 1.107000 4.851000 0.111000
C -2.177000 0.966000 -0.950000	C -4.794000 0.480000 1.968000	H 3.435000 1.906000 3.287000	H 2.028000 3.455000 0.714000
C 1.695000 -2.230000 0.793000	C -4.739000 1.929000 -0.048000	H 2.709000 -2.517000 -2.535000	
C -4.796000 -0.568000 -0.266000	C 1.029000 3.815000 0.442000	H 1.410000 -3.394000 -1.704000	
C 3.904000 -0.256000 0.797000	H -2.224000 1.282000 1.170000	H 3.089000 -3.616000 -1.184000	

C Epoxyazadiradione.xyz

68	C -4.678000 -2.130000 -0.767000	H -2.543000 -0.335000 -1.304000	H 0.203000 1.859000 2.454000
00000001	C -3.502000 -3.017000 -0.705000	H -0.414000 -0.946000 -0.712000	H 2.836000 1.117000 2.475000
C 1.179000 0.810000 0.327000	C 0.406000 -1.861000 1.038000	H 0.922000 1.817000 -1.733000	H 3.611000 -0.488000 2.492000
C 1.587000 1.380000 -1.005000	O -1.069000 2.911000 0.475000	H 3.962000 1.148000 0.343000	H 1.920000 -0.326000 2.968000
C -0.281000 0.598000 0.768000	C -0.509000 3.815000 -0.418000	H -1.041000 1.505000 -1.077000	H -1.824000 -2.139000 2.526000
O 1.731000 2.202000 0.275000	O 3.212000 0.485000 -2.561000	H -1.536000 -3.394000 -0.084000	H -3.519000 -1.961000 2.076000
C 2.297000 -0.155000 0.805000	O 5.908000 -2.420000 -1.173000	H -3.004000 1.273000 1.256000	H -2.574000 -0.534000 2.543000
C -2.121000 -1.314000 0.525000	C 4.842000 -1.545000 -1.404000	H -3.329000 1.865000 -0.374000	H -4.406000 -0.125000 -2.619000
C 2.862000 0.707000 -1.402000	C 5.556000 -1.227000 0.712000	H 2.548000 -2.303000 0.953000	H -5.030000 1.189000 -1.591000
C -0.609000 -0.896000 0.372000	C 6.324000 -2.195000 0.144000	H 1.846000 -1.765000 -0.574000	H -6.040000 -0.241000 -1.929000
C -2.971000 -0.280000 -0.290000	O -5.802000 -2.566000 -1.066000	H -3.657000 -4.013000 -1.104000	H -5.077000 -0.697000 1.648000
C 3.532000 0.243000 -0.108000	O -0.365000 3.581000 -1.615000	H 0.143000 -2.899000 0.811000	H -6.407000 -0.598000 0.478000
C -1.256000 1.521000 -0.007000	C -0.387000 0.957000 2.277000	H 0.378000 -1.755000 2.127000	H -5.425000 0.844000 0.836000
C -4.477000 -0.627000 -0.479000	C 2.681000 0.050000 2.282000	H 4.431000 -1.531000 -2.393000	H 0.043000 5.884000 -0.371000
C -2.340000 -2.665000 -0.139000	C -2.538000 -1.487000 2.014000	H 5.665000 -0.833000 1.709000	H 0.859000 4.825000 0.814000
C -2.727000 1.157000 0.205000	C -5.021000 0.105000 -1.741000	H 7.150000 -2.788000 0.488000	H -0.828000 5.303000 1.096000
C 4.585000 -0.804000 -0.284000	C -5.398000 -0.243000 0.709000	H -0.025000 0.157000 2.920000	
C 1.835000 -1.598000 0.512000	C -0.095000 5.057000 0.326000	H -1.416000 1.183000 2.556000	

D Gedunin.xyz

69	C -1.058000 1.690000 -0.344000	H -0.826000 -0.876000 0.081000	H -2.547000 0.320000 -3.266000
00000001	C -0.242000 0.682000 -1.212000	C -5.436000 0.699000 0.746000	H -3.732000 -0.976000 -3.088000
C -4.070000 -0.032000 0.778000	C 3.615000 -1.352000 0.711000	C -3.356000 0.371000 2.092000	H -0.178000 0.470000 -3.418000
C 3.530000 1.831000 -0.663000	C 4.321000 -2.516000 0.858000	H 1.592000 2.770000 -0.377000	H 0.582000 1.946000 -2.797000
C 2.054000 1.795000 -0.447000	O 4.323000 -2.928000 2.187000	H 4.108000 -1.281000 -1.304000	H -1.173000 1.809000 -2.868000
C 1.261000 0.572000 -0.798000	C 3.592000 -1.956000 2.890000	H 1.296000 -2.108000 -0.023000	H 2.961000 0.519000 -3.026000
C 2.098000 -0.599000 -1.344000	C 3.151000 -0.994000 2.038000	H 1.902000 -2.749000 -1.557000	H 1.534000 -0.433000 -3.477000
C 3.492000 -0.677000 -0.629000	O -5.517000 -2.026000 1.199000	H -0.604000 -2.811000 -1.464000	H 3.069000 -1.244000 -3.173000
O 4.203000 0.631000 -0.655000	C -2.690000 -0.696000 -2.899000	H -0.059000 -1.737000 -2.756000	H 0.639000 0.914000 3.194000
C 1.340000 -1.924000 -1.102000	C -0.256000 1.253000 -2.667000	H -3.998000 -2.322000 -1.396000	H -0.962000 1.506000 3.668000
C -0.089000 -1.865000 -1.669000	C 2.424000 -0.418000 -2.853000	H -2.251000 -1.822000 1.044000	H 0.473000 2.568000 3.860000
C -0.896000 -0.726000 -1.004000	O 4.140000 2.875000 -0.847000	H -3.044000 2.365000 0.076000	H -5.282000 1.783000 0.700000
C -2.403000 -0.820000 -1.379000	O -0.900000 1.387000 1.094000	H -2.724000 2.126000 -1.630000	H -6.006000 0.458000 1.647000
C -3.084000 -2.076000 -0.850000	C -0.181000 2.319000 1.843000	H -0.684000 2.707000 -0.495000	H -6.028000 0.385000 -0.120000
C -3.105000 -2.216000 0.497000	C 0.002000 1.801000 3.245000	H 4.860000 -3.140000 0.167000	H -2.372000 -0.083000 2.188000
C -4.404000 -1.601000 0.932000	O 0.206000 3.388000 1.385000	H 3.501000 -2.115000 3.949000	H -3.990000 0.064000 2.933000
C -3.319000 0.308000 -0.616000	O 1.612000 0.846000 0.638000	H 2.566000 -0.130000 2.279000	H -3.225000 1.455000 2.137000
C -2.577000 1.681000 -0.640000	H -4.167000 0.424000 -1.302000	H -2.052000 -1.371000 -3.477000	

Supplementary 10. A-D. Co-ordinates of structure optimized ligands (basic limonoids).

References

1. Haldar, S., Phapale, P. B., Kolet, S. P., and Thulasiram, H. V. (2013) Expedient preparative isolation, quantification and characterization of limonoids from Neem fruits. Anal Methods. 5, 5386–5391
