# Supplementary material for: Basic Limonoid modulates Chaperone-mediated Proteostasis and dissolve Tau fibrils
Source: Sci Rep. 2020 Mar 4;10:4023. doi: 10.1038/s41598-020-60773-1 (PMC7055235; doi:10.1038/s41598-020-60773-1)
Supplement: Supplementary file 2 — Supplementary Information 2. [file 41598_2020_60773_MOESM2_ESM.pdf]

ANCILLARY FILE

# Basic Limonoid modulates Chaperone-mediated proteostasis and dissolve Tau fibrils

**Nalini V. Gorantla,[a],[c] Rashmi Das,[a],[c] Hariharakrishnan Chidambaram,[a],[c] Tushar Dubey,[a],[c Fayaj A. Mulani,[b],[c]] Hirekodathakallu V. Thulasiram,*,[b],[c] and Subashchandrabose Chinnathambi**,[a],[c]**

[a]Neurobiology Group, Division of Biochemical Sciences, CSIR-National Chemical Laboratory, Dr. Homi Bhabha Road, 411008 Pune, India.

[b]Division of Organic Chemistry, CSIR-National Chemical Laboratory, Dr. Homi Bhabha Road, 411008 Pune, India.

[c]Academy of Scientific and Innovative Research (AcSIR), 411008 Pune, India.

[§]To whom correspondence should be addressed**:** Total extraction and isolation of Limonoids: **Prof. Hirekodathakallu V. Thulasiram**. Email: hv.thulasiram@ncl.res.in **Prof. Subashchandrabose Chinnathambi**, Neurobiology group, Division of Biochemical Sciences, CSIR-National Chemical Laboratory (CSIR-NCL), Dr. Homi Bhabha Road, 411008 Pune, India, Telephone: +91-20-25902232, Fax. +91-20-25902648. Email: s.chinnathambi@ncl.res.in

# Ancillary Figure

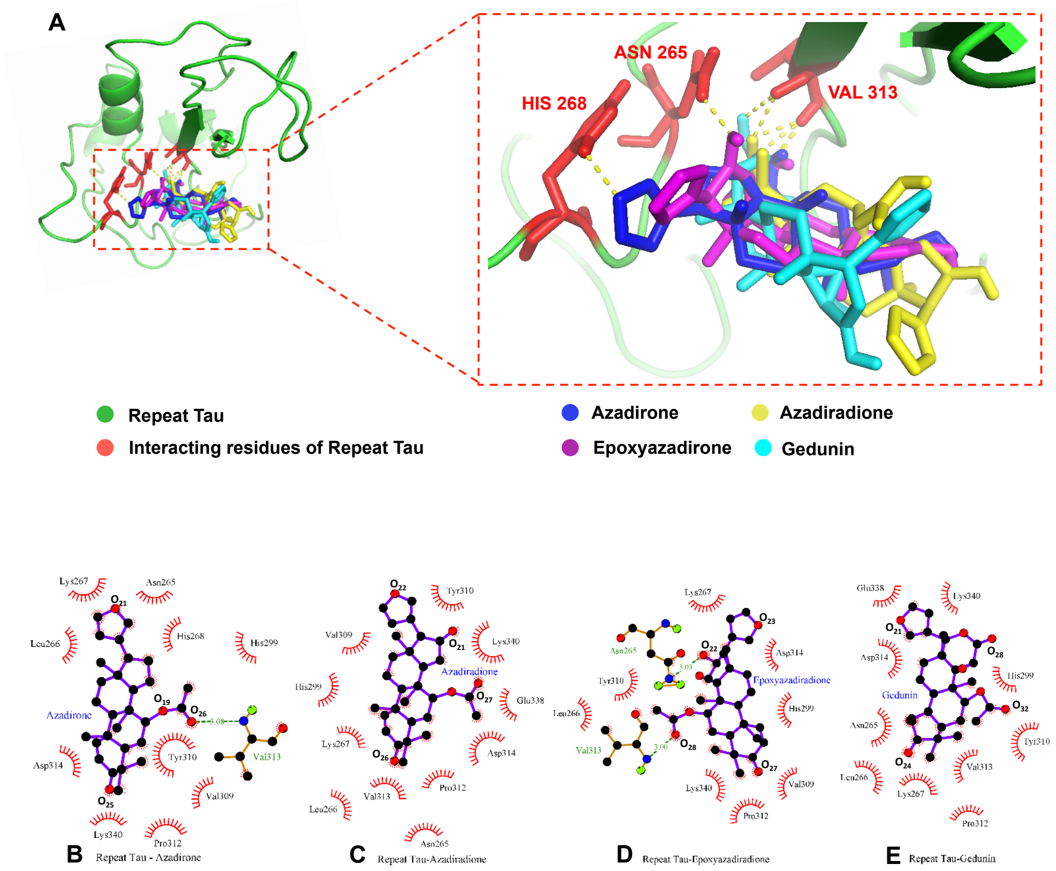


**Figure A1. Molecular Interaction of basic limonoids with Tau model for repeat region (R1-R4). A** shows the binding site for the ligands and the hydrogen bond interactions with the Tau residues. **B-E** represents the 2D-graph for hydrogen bond and hydrophobic interactions of the ligands with the Tau model. **B** Azadirone, **C** Azadiradione, **D** Epoxyazadiradione, and **E** Gedunin. (Repeat Tau model built by Sonawane *et al*. was taken for the docking studies).

# Ancillary Table

| Interacting residues of Tau | Epoxy azadiradione | Azadirone | Gedunin | Azadiradione |
|---|---|---|---|---|
| | -7.34 kcal/mol | -7.28 kcal/mol | -6.62 kcal/mol | -6.60 kcal/mol |
| **Asn265** | 🔵 | 🔴 | 🔴 | 🔴 |
| **Leu266** | 🔴 | 🔴 | 🔴 | 🔴 |
| **Lys267** | 🔴 | 🔴 | 🔴 | 🔴 |
| **His268** | - | 🔴 | 🔴 | - |
| **His299** | 🔴 | - | 🔴 | 🔴 |
| **Val309** | 🔴 | 🔴 | 🔴 | 🔴 |
| **Tyr310** | 🔴 | 🔴 | 🔴 | 🔴 |
| **Pro312** | 🔴 | 🔴 | 🔴 | 🔴 |
| **Val313** | 🔵 | 🔵 | 🔴 | 🔴 |
| **Asp314** | 🔴 | 🔴 | 🔴 | 🔴 |
| **Glu338** | - | - | 🔴 | 🔴 |
| **Lys340** | 🔴 | 🔴 | 🔴 | 🔴 |

**Residues with Hydrogen bond interactions** 🔵

**Residues with Hydrophobic interactions** 🔴

**Non-interacting residues** -

**Table.1** Tau residues involved in hydrogen bond and hydrophobic interactions.

**References:**

Sonawane, Shweta Kishor, et al. "Baicalein suppresses Repeat Tau fibrillization by sequestering oligomers." Archives of Biochemistry and Biophysics 675 (2019): 108119.
