# Supplementary material for: Basic Limonoid modulates Chaperone-mediated Proteostasis and dissolve Tau fibrils
Source: Sci Rep. 2020 Mar 4;10:4023. doi: 10.1038/s41598-020-60773-1 (PMC7055235; doi:10.1038/s41598-020-60773-1)
Supplement: Supplementary file 3 — Supplementary Information 3. [file 41598_2020_60773_MOESM3_ESM.pdf]

Ancillary Figure 1

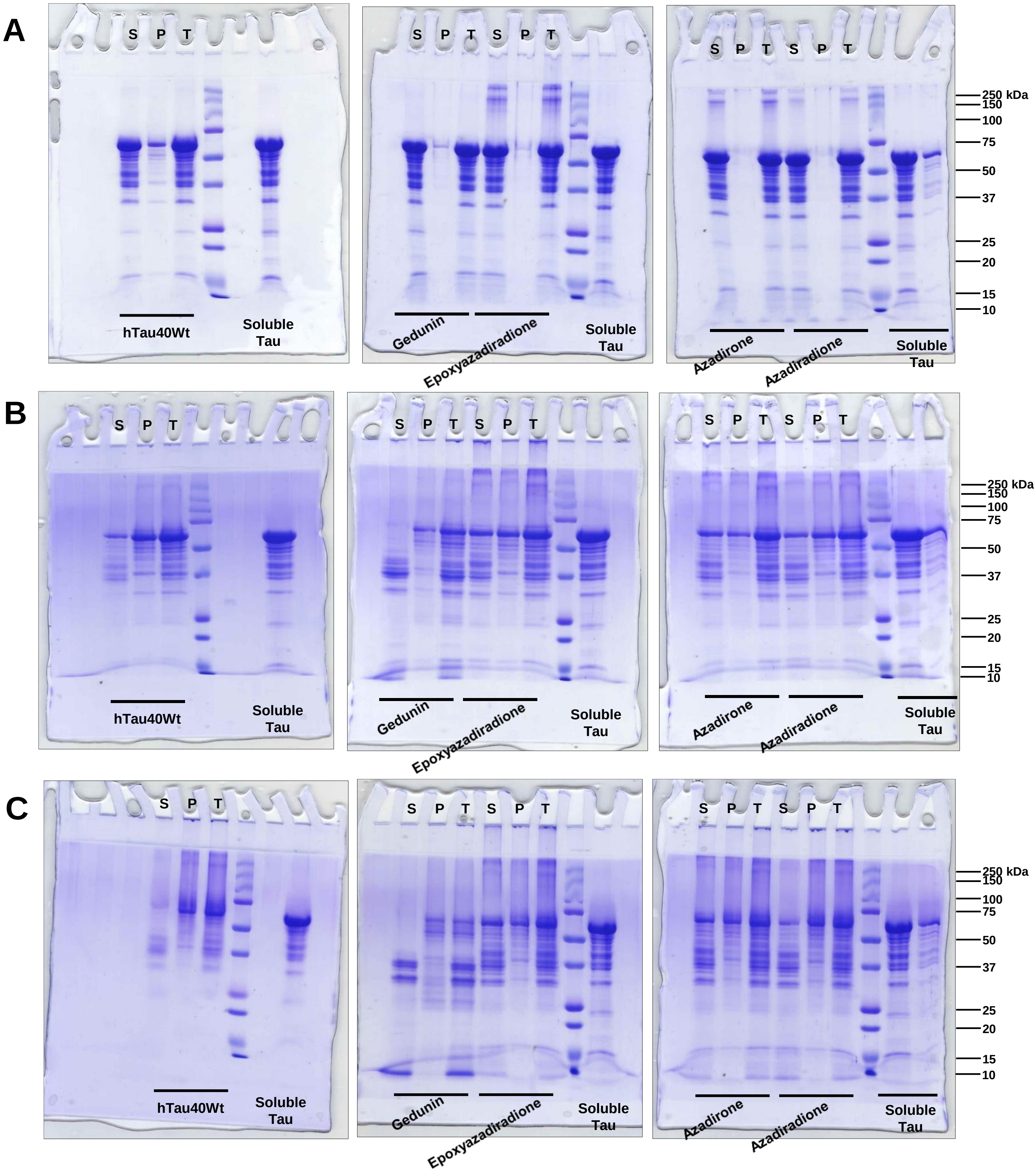

The above SDS-PAGE are the complete gels i.e., without cropping. These gels corresponds to SDS-PAGE at A. 0 hour, B. 24 hours and C. 120 hours of aggregation inhibition assay. In the main draft they are represented as figure 1F, G and H respectively.

Ancillary Figure 2

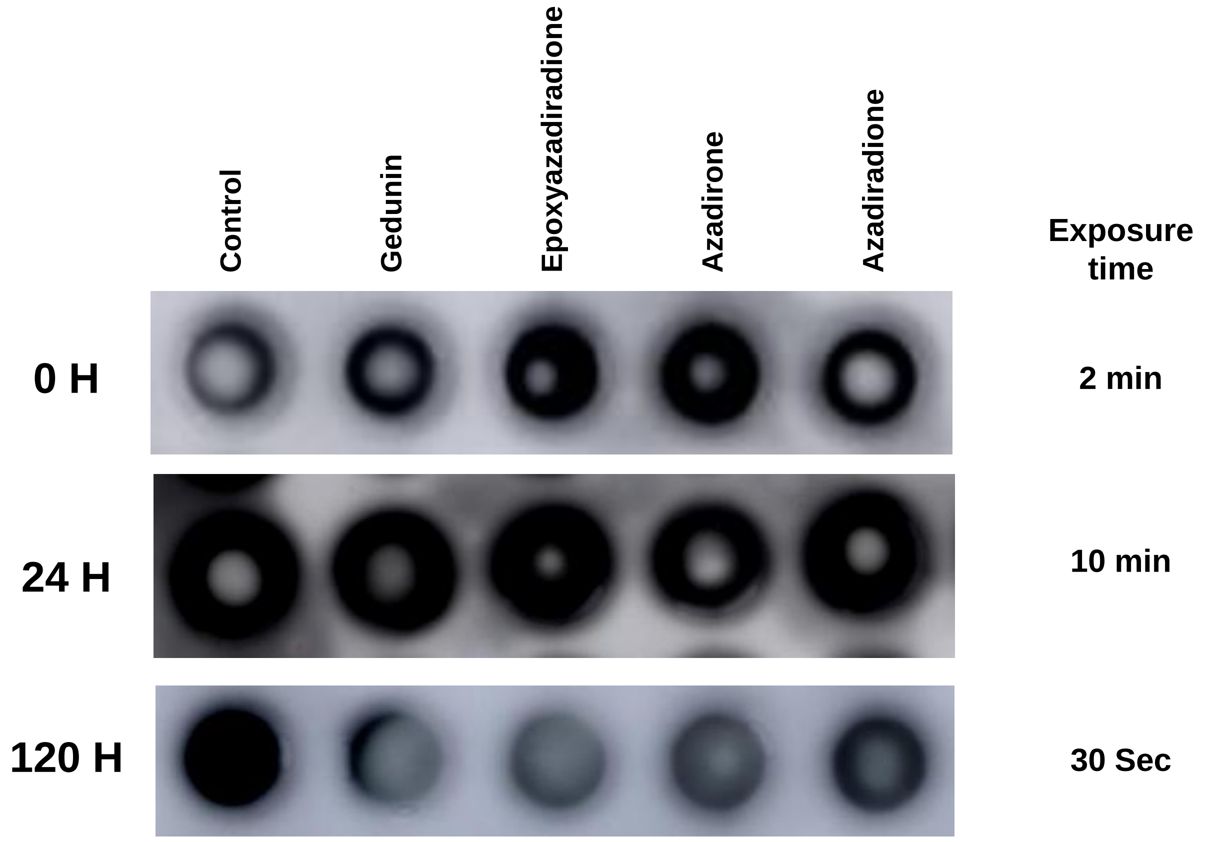

The above are the blots for filter trap assay at 0, 24 and 120 hours of aggregation inhibition assay, with their corresponding exposure time. This has been represented as figure. 1J in the main draft .

Ancillary Figure 3

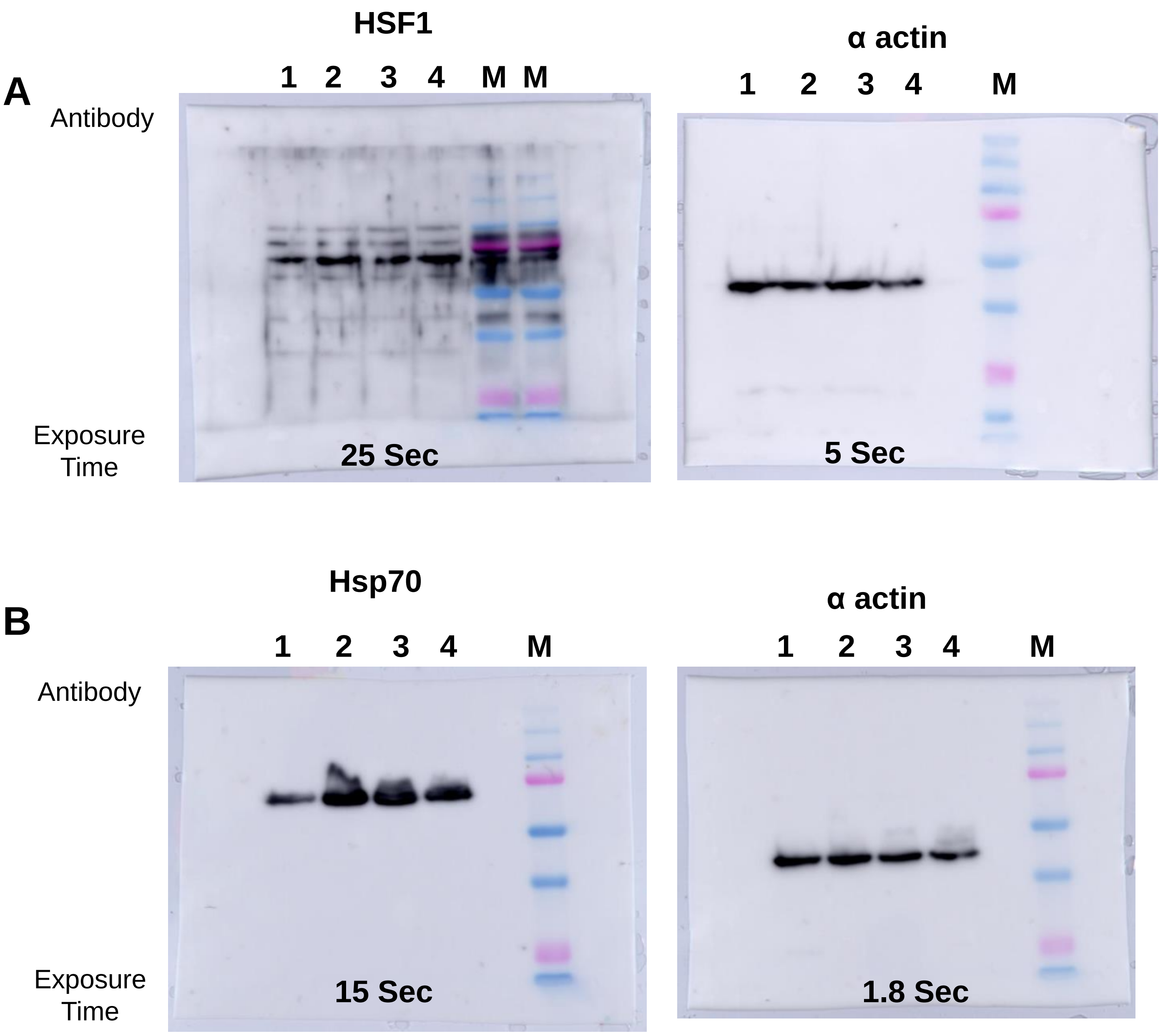

1. Cell control
2. Tau aggregates
3. Epoxyazadiradione
4. Epoxyazadiradione+ Tau aggregates

M. Marker

The above are the western blots of A. HSF1, B. Hsp70 and their respective loading controls i.e., α actin. The corresponding exposure time for each blots are also mentioned. These were represented as figure. 6A and 8A respectively.

Ancillary Figure 4

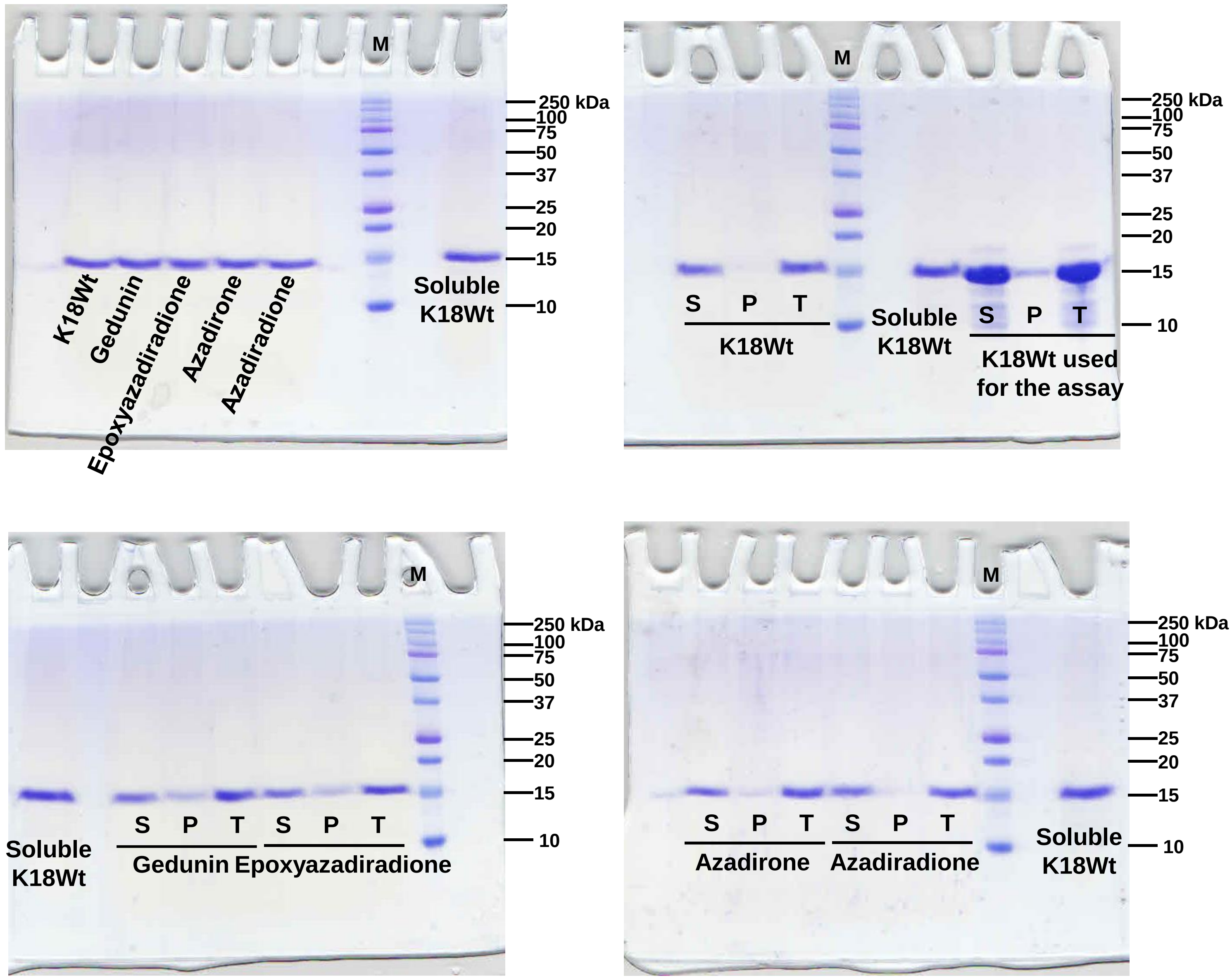

The above SDS-PAGE are the complete gels for K18wt represented as S4B and C in the supplementary file.
